# Protein Kinase C and Extracellular Signal-Regulated Kinase Regulate Movement, Attachment, Pairing and Egg Release in *Schistosoma mansoni*

**DOI:** 10.1371/journal.pntd.0002924

**Published:** 2014-06-12

**Authors:** Margarida Ressurreição, Paulu De Saram, Ruth S. Kirk, David Rollinson, Aidan M. Emery, Nigel M. Page, Angela J. Davies, Anthony J. Walker

**Affiliations:** 1 Molecular Parasitology Laboratory, School of Life Sciences, Kingston University, Kingston upon Thames, Surrey, United Kingdom; 2 Wolfson Wellcome Biomedical Laboratories, Life Sciences Department, Natural History Museum, London, United Kingdom; University of Melbourne, Australia

## Abstract

Protein kinases C (PKCs) and extracellular signal-regulated kinases (ERKs) are evolutionary conserved cell signalling enzymes that coordinate cell function. Here we have employed biochemical approaches using ‘smart’ antibodies and functional screening to unravel the importance of these enzymes to *Schistosoma mansoni* physiology. Various PKC and ERK isotypes were detected, and were differentially phosphorylated (activated) throughout the various *S. mansoni* life stages, suggesting isotype-specific roles and differences in signalling complexity during parasite development. Functional kinase mapping in adult worms revealed that activated PKC and ERK were particularly associated with the adult male tegument, musculature and oesophagus and occasionally with the oesophageal gland; other structures possessing detectable activated PKC and/or ERK included the Mehlis' gland, ootype, lumen of the vitellaria, seminal receptacle and excretory ducts. Pharmacological modulation of PKC and ERK activity in adult worms using GF109203X, U0126, or PMA, resulted in significant physiological disturbance commensurate with these proteins occupying a central position in signalling pathways associated with schistosome muscular activity, neuromuscular coordination, reproductive function, attachment and pairing. Increased activation of ERK and PKC was also detected in worms following praziquantel treatment, with increased signalling associated with the tegument and excretory system and activated ERK localizing to previously unseen structures, including the cephalic ganglia. These findings support roles for PKC and ERK in *S. mansoni* homeostasis, and identify these kinase groups as potential targets for chemotherapeutic treatments against human schistosomiasis, a neglected tropical disease of enormous public health significance.

## Author Summary

Parasitic blood flukes, also called schistosomes, cause human schistosomiasis, a neglected tropical disease and major public health problem in developing countries, especially sub-Saharan Africa. Sustainable control of schistosomiasis is difficult, mainly because the complex life cycle of the parasite involves a freshwater snail host, and the ability of the parasite to evade the immune response of the human host and to survive for many years. Little is yet known about the cellular mechanisms in schistosomes and how they regulate parasite homeostasis, development and behaviour. In this paper, the nature of intracellular signalling by protein kinases C (PKCs) and extracellular signal-regulated kinases (ERKs) in schistosomes is studied and these proteins are found to be vital for the coordination of processes fundamental to parasite survival, such as muscular activity and reproductive function. Our results contribute to an understanding of molecular events regulating schistosome function and identify PKCs and ERKs as possible targets for the development of new chemotherapeutic treatments against schistosomiasis.

## Introduction

Protein kinases C (PKCs) and extracellular signal-regulated kinases/mitogen-activated protein kinases (ERKs/MAPKs) are signalling enzymes that play a critical role in regulating cellular processes, such as gene expression, the cell cycle, growth, development and differentiation, cellular motility, survival and apoptosis [1], [2]. PKC/ERK signalling occurs in response to various stimuli, including ligands that bind receptor tyrosine kinases (RTKs) and G-protein coupled receptors (GPCRs) [1], [2]. Putative PKCs and ERKs exist in kinomes of the blood flukes *Schistosoma mansoni* [3], [4], *S. japonicum* [5] and *S. haematobium* [6]. These parasites cause human schistosomiasis, a neglected tropical disease (NTD) characterised by inflammatory granulomatous reactions in the host organs that occur in response to entrapped eggs from adult female worms [7]. The global significance of human schistosomiasis is huge; more than 200 million people have the disease, and 0.8 billion are at risk of infection [8], [9]. Chemotherapy relies upon praziquantel (PZQ) treatment, but this compound kills adult worms and not juvenile stages, and does not prevent re-infection [10], [11]; emergence of drug resistant strains is also possible [12]. Although anti-schistosome vaccine targets exist [13], some worms exhibit antigenic polymorphism, making targeting difficult [14]. Thus, there is considerable interest in identifying new anti-schistosome drug targets, and the protein kinases are potential candidate molecules [15], [16].

In humans 10 PKCs exist, including PKCβ_I_ and PKCβ_II_, which arise from alternate gene splicing [17]. PKCs comprise a C-terminal serine/threonine kinase domain linked through a flexible hinge segment to an N-terminal regulatory region that contains diacylglycerol (DAG)-sensitive and Ca^2+^-sensitive domains [18]. Conventional PKCs (cPKCs: PKCα, PKCβI/βII, and PKCγ) are DAG sensitive and Ca^2+^ responsive; novel PKCs (nPKCs: PKCδ, PKCε, PKCη, and PKCθ) are DAG sensitive but Ca^2+^ insensitive; and atypical PKCs (aPKCs: PKCζ and PKCι, or λ-murine) are insensitive to both DAG and Ca^2+^ [18]. Phosphorylation on specific amino acid residues is also crucial to PKC activation [19]. Previously, a DAG/Ca^2+^-dependent PKC-like activity and a phospholipid/phorbol ester sensitive kinase activity were detected in adult *S. mansoni* homogenates [20], [21], and a PKC (SmPKC1) homologous to human PKCβ was characterised molecularly [22]. Previously, we identified four putative PKCs in the *S. mansoni* genome with homology to human PKCs, particularly within functional domains [23]; two proteins were similar to human cPKCβI, one to nPKCε and one to aPKCζ [23], with PKCε also being designated PKCη [4]. Using phospho-specific antibodies, we showed that activated PKCβ associated with the neural mass, tegument, ciliated plates and germinal cells of miracidia, and that PKC activation restricted development to mother sporocysts that parasitize the snail intermediate host [23].

MAPK pathways exist in all eukaryotes, with components being conserved among yeast, invertebrates and mammals [24]–[29]. The ERK pathway features Ras as a monomeric G-protein, Raf as a MAPKKK, MAPK/ERK Kinase (MEK) as a MAPKK, and ERK as a MAPK, the last three forming a hierarchical kinase cascade [30]. Humans and many other organisms express ERK1 and ERK2 (p44 and p42 MAPK) to varying extents in tissues and more than 150 ERK1/2 substrates exist [2], including cytosolic, membrane, nuclear and cytoskeletal proteins [30]. Phosphorylation of ERK1/2 on threonine and tyrosine resides within the Thr-Glu-Tyr (TEY) motif in the activation loop is essential for activation. In *S. mansoni*, Ras GTPase activator protein- (Ras-GAP) and ERK1/2-like proteins have been detected [31], and a Ras homologue has been characterised [32]. Activation of the *S. mansoni* epidermal growth factor receptor (EGFR; SER) by human EGF leads to ERK2 phosphorylation in *Xenopus* oocytes [33], and hypothetical ERK pathways for *S. mansoni* and *S. japonicum* have been reconstructed *in silico* [3], [4], [34], supporting that ERK signalling is intact in schistosomes.

Identifying signalling molecules that play a fundamental role in cellular communication and function of schistosomes represents one of the great challenges of the schistosome post-genomic era [16]. The aims of the current study were to characterise global PKC and ERK signalling in *S. mansoni* and to determine whether PKC and ERK are critical to schistosome function. We demonstrated several PKC and ERK isotypes, profiling activities in different life-stages. We determined PKC and ERK responses to kinase activators and inhibitors in adult worms, and determined the localization of active kinases in intact worms *in situ* through functional kinase mapping. Finally, we showed physiological roles for PKC and ERK in schistosomes through the modulation of kinase activities using pharmacological agents. The findings, together with those also presented for the effects of PZQ on PKC/ERK signalling, establish a role for PKC and ERK in worm homeostasis and behaviour, and identify these kinase groups as potential anti-schistosome drug targets.

## Materials and Methods

### Ethics statement

Laboratory animal use was within a designated facility, regulated under the terms of the UK Animals (Scientific Procedures) Act, 1986, complying with all requirements therein; regular independent Home Office inspections occurred. The Natural History Museum Ethical Review Board approved experiments involving mice and work was carried out under Home Office project license 70/6834.

### Antibodies and reagents

Anti-phospho antibodies (Ab) used to detect phosphorylated PKC and ERK in *S. mansoni* were anti-phospho-PKC (pan) (βII Ser660) rabbit Ab (#9371), anti-phospho-PKC (pan) (ζ Thr410) (190D10) rabbit mAb (#2060), immobilized anti-phospho p44/p42 MAPK (ERK1/2) (Thr202/Tyr204) (D13.14.4E) XP rabbit mAb (#3510), and anti-phospho-p44/42 MAPK (ERK1/2) (Thr202/Tyr204) rabbit mAb (#9101) (Cell Signalling Technology, New England Biolabs). The p44/p42 MAPK (ERK1/2) (non-radioactive) immunoprecipitation/kinase assay kit, phorbol 12-myristate 13-acetate (PMA), cell lysis buffer, RIPA buffer, lambda phosphatase, and anti-rabbit horseradish peroxidase (HRP)-linked secondary antibodies were also purchased from Cell Signalling Technology. The Omnia S/T peptide 8 kinase assay kit (KNZ1081), RPMI-1640 medium, foetal bovine serum, antibiotics/antimycotics, and anti-rabbit Alexa Fluor 488 secondary antibodies were purchased from Invitrogen. Pre-cast Precise 10% polyacrylamide gels, West Pico chemiluminescence substrate, Restore Western blot stripping buffer and Halt protease/phosphatase inhibitor cocktail were from Pierce, whereas nitrocellulose membrane was from GE Health. U0126, GF109203X, and PKC catalytic subunit derived from rat brain were purchased from Merck. Vectashield mounting medium was from Vector Laboratories.

### Bioinformatic characterisation of *S. mansoni* PKCs and ERKs

PKC and ERK gene candidates were identified from the *S. mansoni* genome assembly, relying on existing annotations (http://www.genedb.org/genedb/smansoni), Andrade et al. [4], and our existing analysis [23]. Protein sequences were assessed for similarity with other organisms using pBLAST (http://www.uniprot.org). The detection site of the anti-phospho antibodies was identified within the putative *S. mansoni* PKC and ERK sequences and was aligned to human PKC and ERKs using MUSCLE (www.ebi.ac.uk/Tools/msa/muscle).

### Isolation of schistosomes

The Belo Horizonte strain of *S. mansoni* was maintained in *Biomphalaria glabrata* and albino female mice (BKW strain). Miracidia and *in vitro* transformed sporocysts were collected, concentrated, and processed for Western blotting as previously described [35], [36]. Briefly, cercariae were collected after they emerged from patent snails and were transferred to 15 ml conical tubes and cooled on ice for 15 min prior to centrifugation (200× *g*). Concentrated cercariae were transferred to 1.5 ml microfuge tubes, pelleted by pulse centrifugation, and homogenized on ice in 25 µl RIPA buffer containing Halt protease/phosphatase inhibitors. An aliquot was removed for protein quantification (Bradford assay with Bovine serum albumin (BSA) as protein standard), 5× SDS-PAGE sample buffer added and samples heated at 95°C for 5 min. Extracts were then either electrophoresed immediately or stored at −80°C. Adult worms were collected by hepatic portal perfusion of mice 40–42 days post infection and were gently washed with pre-warmed RPMI 1640 and either frozen in liquid nitrogen and stored at −80°C for immunoprecipitation/kinase assay, fixed on ice in acetone and stored at 4°C for immunohistochemistry, or placed in RPMI 1640 at 37°C.

### Exposure of adult *S. mansoni* to GF109203X, U0126 or PMA to assess effects on kinase phosphorylation

Following equilibration at 37°C for 30 min in RPMI 1640, live adult worms were treated for various durations (0, 15, 30, 60, or 120 min) with either 20 µM GF109203X, 1 µM PMA, 1 µM U0126, dimethyl sulphoxide (DMSO; vehicle for PMA or U0126, 0.2% or 0.1%, respectively), or were left untreated (RPMI 1640 only). In addition, live adult worms were also incubated in 1 µM PMA or DMSO for 24 h at 37°C/5% CO_2_ in RPMI containing glutamine, glucose, antibiotic/antimycotic mixture (100 U penicillin, 100 µg streptomycin and 0.25 µg amphotericin B/ml) and 10% FBS. GF109203X is a PKC inhibitor that competes for the ATP binding site and therefore only catalytically inactive proteins are inhibited. The effect of GF109203X on PKC was thus established by pre-incubating worms in 20 µM GF109203X or RPMI 1640 for 120 min prior to exposure to 1 µM PMA for 30 min at 37°C. The MEK inhibitor U0126 inhibits active and inactive MEK1/2 blocking ERK phosphorylation. Immediately after treatment, medium was removed and worms were either homogenized in 30 µl RIPA buffer and processed for Western blotting or were acetone-fixed for immunohistochemistry in similar ways to that for cercariae (above).

### *S. mansoni* PKC and ERK immunoprecipitation and kinase activity assay

Perfused adult worms (100 or 20 pairs for PKC or ERK assay, respectively) were transferred to microfuge tubes and washed twice with RPMI 1640 at 37°C. Worms were then exposed to 1 µM PMA or DMSO (vehicle) in RPMI 1640 for 30 min at 37°C prior to being snap frozen in liquid nitrogen and stored at −80°C. When required, worm pairs were defrosted and homogenized on ice in 100 µl cell lysis buffer with Halt protease/phosphatase inhibitor cocktail. The homogenate was then centrifuged for 15 min at 4°C, the supernatant recovered, and the remaining pellet re-homogenized in 50 µl cell lysis buffer with inhibitors and centrifuged for a further 10 min. Supernatants were pooled and equal quantities of protein from each sample incubated overnight at 4°C in either anti-phospho PKC (pan) (ζ Thr410), anti-phospho PKC (pan) (β_II_ Ser660) antibodies, or immobilized anti-phospho p44/p42 MAPK (ERK1/2) (Thr202/Tyr204) (D13.14.4E) XP primary antibodies (each at 1/25 dilution). The next day, 50 µl protein A agarose beads were added to homogenates and agitated for 5 min at 4°C except when the pre-immobilized anti-phospho p44/p42 MAPK (ERK1/2) antibody was used; such rapid immunocapture reduces non-specific binding while permitting efficient immunocomplex adsorption [35], [37]. Following brief centrifugation, samples were washed twice each in 500 µl ice-cold lysis buffer and 500 µl ice-cold kinase buffer (25 mM Tris (pH 7.5), 5 mM β-glycerolphosphate, 2 mM DTT, 0.1 mM Na_3_VO_4_ and 10 mM MgCl_2_). Negative control immunoprecipitations lacking primary antibodies were also performed.

The Omnia (Ser/Thr 8 peptide) PKC assay was performed as per the manufacturer's instructions to detect immunoprecipitated PKC activity. Master mix, containing ATP, Ser/Thr 8 substrate, DTT, kinase buffer and water was added to individual wells of black 96-well microtitre plates (Nunc) and reactions started by adding immunocomplex. Accumulation of phosphorylated substrate was measured (excitation 355 nm, emission 460 nm) every 30 s for up to 200 min at 30°C using a FLUOstar OPTIMA (BMG Labtech) microplate reader. Positive control reactions contained 2 ng recombinant human PKC; negative controls either lacked the substrate or comprised samples prepared without the immunoprecipitation antibody. Immunoprecipitated ERK activity was detected using the p44/p42 MAPK (ERK1/2) assay kit. Immunocomplexes were re-suspended in 20 µl kinase buffer supplemented with 200 µm ATP and 2 µg Elk-1 fusion protein and incubated for 30 min at 30°C. Reactions were terminated with 10 µl 3× SDS-PAGE sample buffer and samples heated at 95°C for 5 min in preparation for SDS-PAGE and Western blotting with anti-phospho Elk-1 antibodies to reveal ERK1/2 activity. Negative controls comprised samples without immunoprecipitation antibody.

### *In vitro* culture of adult *S. mansoni* with GF109203X, U0126 or PMA

Adult worms were placed in individual wells of a 24-well culture plate (Nunc; 3–6 worm pairs per well) in 1.5 ml RPMI 1640 containing glutamine, glucose, antibiotic/antimycotic mixture and 10% FBS at 37°C/5% CO_2_ [22] for 1 h to equilibrate. To determine effects of PKC inhibition/activation or ERK inhibition on adult worms, 1 µM, 5 µM, 20 µM or 50 µM GF109203X or U0126, or 1 µM PMA were added to cultures. Control groups containing RPMI 1640 only or 0.5% DMSO (vehicle for PMA/U0126) were also included and all media and components were replenished daily. Worms were observed at various times over 96 h using an Olympus SZ54045 binocular dissecting microscope and avi-format movies captured using a JVC TK-1481 composite colour video camera linked to Studio Launcher Plus for Windows software. Worm behaviour including pairing status and ventral sucker attachment to the base of the culture plate was determined. Egg release by worms was also enumerated. Detailed analysis of worm movement was done using image J [38] (http://rsbweb.nih.gov/ij/); the diameter of the well in pixels was calibrated to mm and the distance travelled by the posterior tip of each worm in 10 s was manually tracked and measured enabling translation into speed of movement (velocity) of pixels/s to mm/s. Worm coiling (Figure 10A) was determined by counting the number of coils that persisted during 10 s visualization. Results are representative of four independent experiments with a minimum of three replicates each; 30 or more parasites per treatment were scored, except for DMSO controls (n = 24).

### Effect of praziquantel on adult *S. mansoni* PKC and ERK phosphorylation

Racemic PZQ powder (Shin Poong Pharmaceutical) was dissolved in DMSO. Freshly perfused adult worm pairs were incubated in RPMI 1640 containing 0.2 µg/ml PZQ, 0.1% DMSO (vehicle), or RPMI 1640 alone at 37°C for 15, 30 or 120 min. Next, worms were either processed for Western blotting or immunohistochemistry as detailed above. The final concentration of PZQ used was similar to a study [39] that reported 0.2 µg/ml PZQ to be the lowest concentration needed to induce maximal muscular contraction in worms, a phenotype observed with PZQ treatment.

### SDS-PAGE and western blotting

Equal amounts of sample protein (12 µg) were separated on 10% Precise SDS–PAGE gels, semi-dry transferred to nitrocellulose membranes and stained with Ponceau S to confirm homogeneous transfer. After blocking for 1 h in 5% non-fat dried milk, membranes were washed three times in 0.1% Tween-Tris-buffered saline (TTBS) and incubated in anti-phospho PKC (pan) (ζ Thr410), anti-phospho PKC (pan) (β_II_ Ser660) or anti-phospho p44/p42 MAPK (ERK1/2) (Thr202/Tyr204) antibodies (1∶1000 in TTBS) overnight at 4°C. After TTBS wash, blots were incubated for 2 h at room temperature with HRP-conjugated secondary antibodies (1∶3000 in TTBS) and exposed to West Pico chemiluminescence substrate. Immunoreactive bands were visualized with a GeneGnome imaging system (Syngene) and relative band intensities quantified using GeneTools software. Protein loading was determined by incubating blots with anti-actin antibodies (1∶1000). When necessary blots were stripped with Restore Western blot stripping buffer and incubated in an alternative antibody. In addition, to confirm that the anti-phospho PKC and anti-phospho ERK antibodies detected only the phosphorylated kinases, blots were treated with lambda phosphatase (400 U/ml in TTBS containing 1% BSA and 2 mM MnCl_2_) for 4 h before incubation in primary antibodies; secondary antibody labeling and detection were then performed.

### Immunohistochemistry

Acetone-fixed worms were washed twice with PBS, treated with 100 mM glycine for 15 min and blocked in 10% goat serum for 1 h. After a further wash, worms were incubated in anti-phospho PKC (pan) (ζ Thr410), anti-phospho PKC (pan) (β_II_ Ser660) or anti-phospho p44/p42 MAPK (ERK1/2) (Thr202/Tyr204) antibodies (1∶50 dilution in PBS with 5% BSA) for 3 days on a rotator. Worms were then washed twice in PBS for 1 h each and incubated in Alexa Fluor 488 secondary antibodies (1∶500 in 5% BSA) and 2 µg/ml rhodamine phalloidin for 24 h followed by two 1 h washes in PBS. Next, specimens were mounted on slides in Vectashield. All incubations were carried out at room temperature and washes were done in microfuge tubes. Adult worms were visualized on a Leica TCS SP2 AOBS confocal laser scanning microscope using 20× dry, 40× or 62× oil immersion objectives and images captured. The signal received from negative controls (i.e. worms incubated with secondary antibody only) was negated from that of the positive samples. This was achieved by reducing the power level of the photomultiplier tube, which was then kept constant for all observations.

### Statistical analysis

Statistical comparisons were performed with one-way analysis of variance (ANOVA) using Minitab (version 16). All data were expressed as mean ±SEM, and statistical significance was determined by a Fisher's multiple pair-wise comparison.

## Results/Discussion

### Phosphorylated PKC- and ERK-like proteins in *S. mansoni* life stages

**Figure 1:**
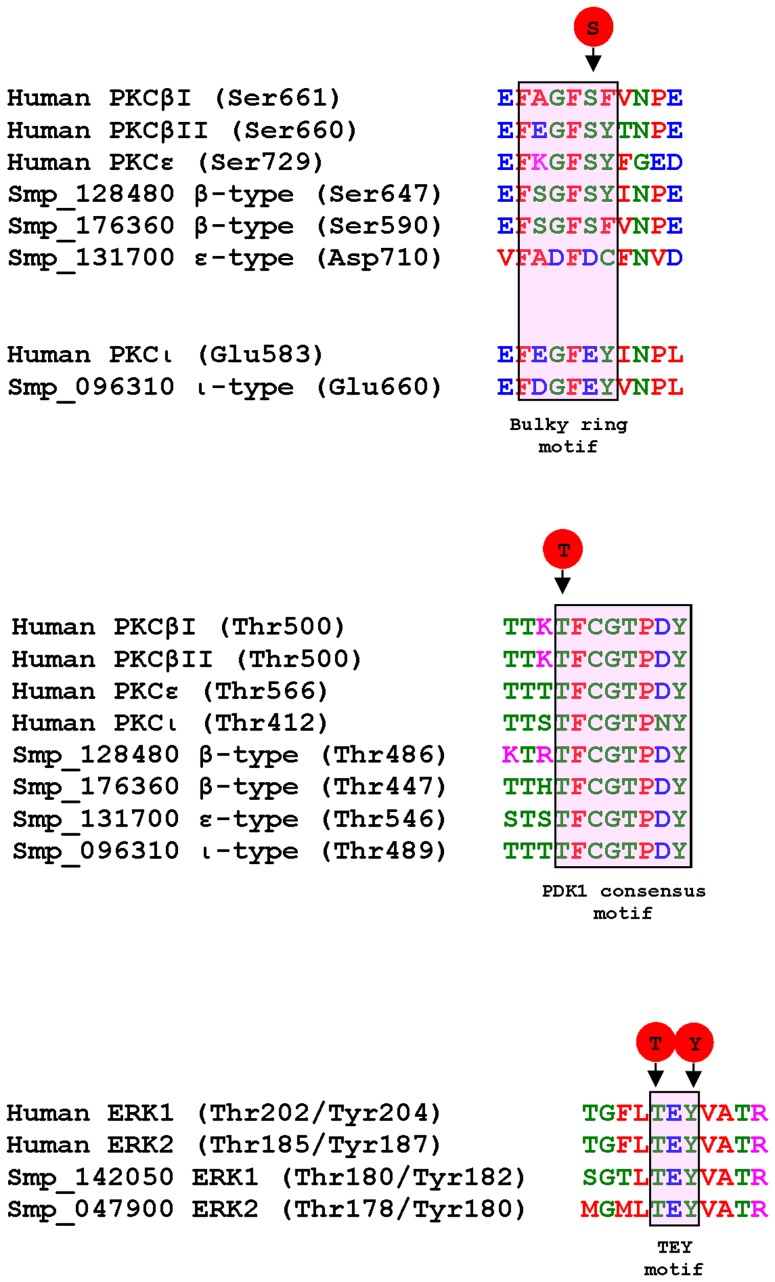
Alignment of antibody recognition sites. Comparison of the amino acid recognition sequences, together with the phosphorylated site, for the three anti-phospho antibodies used between *S. mansoni* PKC and ERK predicted protein sequences obtained from the *S. mansoni* genome database and relevant human protein sequences. The anti-phospho PKC (pan) (βII Ser660) antibodies recognize multiple human PKCs including PKCβI, βII and ε; based on sequence similarity these antibodies are predicted to react with Smp_128480 and Smp_176360 (both β-type PKCs) only when phosphorylated on Ser647 and Ser590, respectively within the bulky ring motif. The *S. mansoni* ε-type PKC (Smp_131700) lacks the conserved Ser phosphorylation site, as does ι-type PKC (Smp_096310) in common with human PKCι. The anti-phospho PKC (pan) (ζ Thr410) antibodies also recognize multiple human PKCs including PKCβI, βII, ε and ι; based on sequence similarity these antibodies are predicted to react with all *S. mansoni* PKCs only when phosphorylated on the conserved Thr residue within the PDK1 consensus motif. The anti-phospho p44/42 MAPK (Thr202/Tyr204) antibodies recognize human ERK1 and ERK2 only when phosphorylated on Thr and Tyr within the conserved TEY motif; based on sequence similarity, these antibodies are predicted to detect *S. mansoni* ERK1 and ERK 2 (Smp_142050 and Smp_047900, respectively) when phosphorylated.

The phospho-specific antibodies used in this study were anti-phospho PKC (pan) (ζ Thr410), anti-phospho PKC (pan) (β_II_ Ser660) and anti-phospho p44/p42 MAPK (ERK1/2) (Thr202/Tyr204). Using the *S. mansoni* genome, four putative PKCs [23] and five putative ERKs displaying homology to human isotypes were identified from full-length sequences. Comparative analysis revealed that, except for Smp_131700 (∼95 kDa PKC), the antibody recognition sequence was conserved in the putative PKCs and ERK1/2, with the key phosphorylation motif retained; similar levels of sequence homology exist between the human isotypes recognized by these antibodies (Figure 1). Such motif conservation is common, because of the critical nature of the phosphorylation site in activation of the respective kinases [1], [2], [18], [40]. As with human PKCs, in *S. mansoni*, the sequence surrounding the phosphorylated Thr residue within the PDK1 consensus motif was more conserved than that surrounding the Ser phosphorylation site in the bulky ring motif (Figure 1).

**Figure 2:**
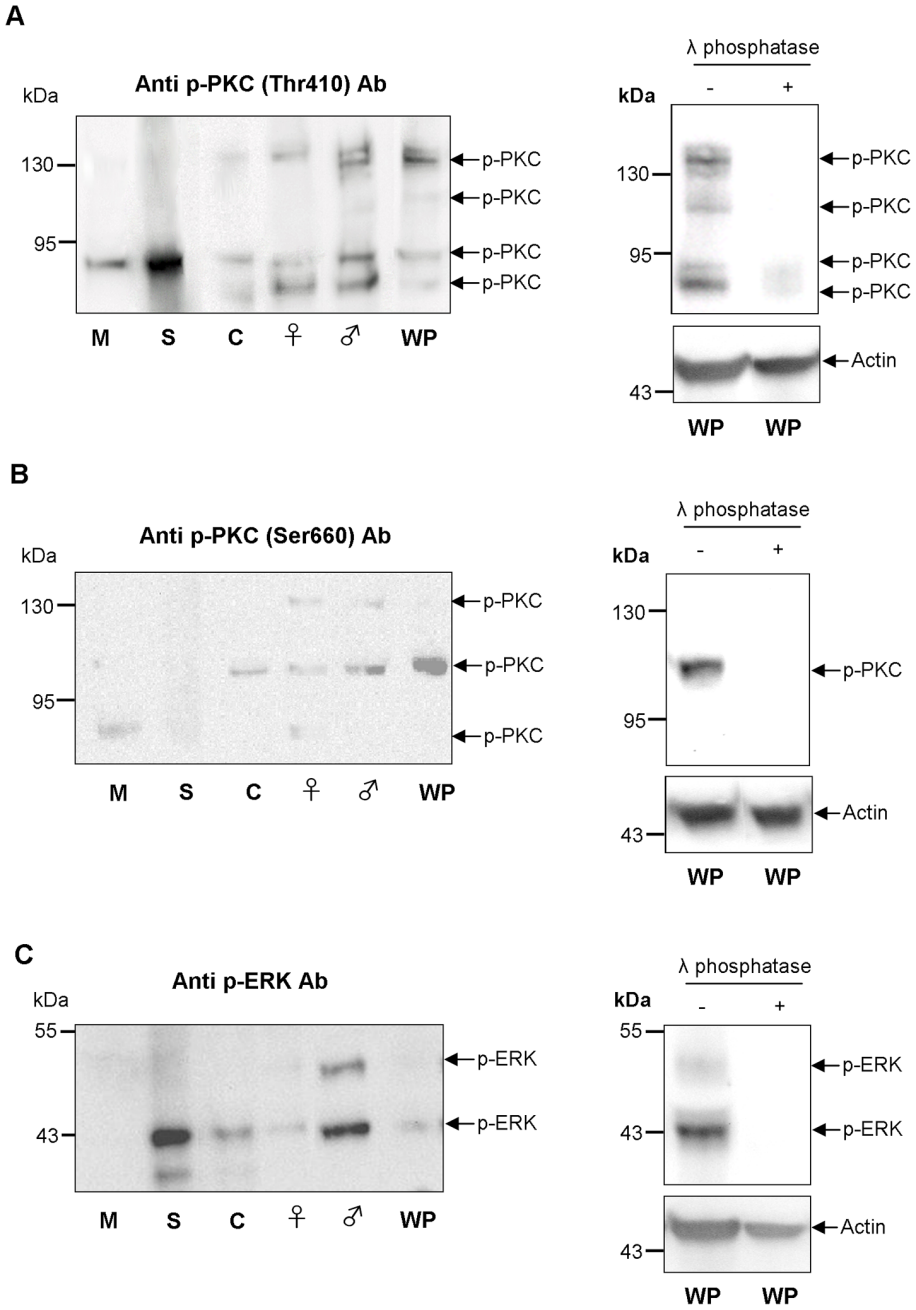
Phosphorylated (activated) PKC- and ERK-like proteins exist in four different life stages of *S. mansoni*. Western blot showing immunoreactive bands detected with (A) anti-phospho PKC (pan) (ζ Thr410), (B) anti-phospho PKC (pan) (βII Ser660), and (C) anti-phospho p44/42 MAPK (ERK1/2) (Thr202/Tyr204) antibodies (Ab) in miracidia (M), mother sporocysts (S), cercariae (C), adult female (♀) and male (♂) worms, and coupled worm pairs (WP) (∼12 µg for each sample). Results are representative of three independent experiments. Lambda phosphatase was also employed to confirm that the antibodies reacted only with the phosphorylated form of each protein; actin was used a loading control.

These antibodies were then used to detect phosphorylated (activated) PKCs and ERK1/2 in homogenates (12 µg protein) of four different life stages (miracidium, sporocyst, cercaria and adult worm) of *S. mansoni* by Western blotting. The anti-phospho PKC (pan) (ζ Thr410) antibodies have been used previously to detect activated PKCs in invertebrates other than *S. mansoni* (e.g. [41]). They detect PKCα, β_I_, β_II_, γ, δ, ε, η, θ, and ζ/ι isotypes only when phosphorylated at a site homologous to Thr410 of human PKCζ (Figure 1) that is critical to catalytic competency and PKC activation [42]. In adult worms and cercariae, three immunoreactive bands were consistently detected, namely an ∼78/∼81 kDa doublet and a ∼132 kDa band with greater immunoreactivity in adult males than cercariae (Figure 2A). A weak band was also seen just above the ∼132 kDa band; however, this was not quantified in later experiments with worm pairs, as it was not always evident. Occasionally, a weak immunoreactive band was also detected at ∼116 kDa in male worms and worm pairs (Figure 2A). In sporocysts and miracidia, a single ∼81 kDa band was detected which appeared highly phosphorylated in sporocysts compared to the other life stages studied (Figure 2A).

Previously, we validated anti-phospho PKC (pan) (β_II_ Ser660) antibodies to detect phosphorylated (activated) PKC in *S. mansoni* miracidia and sporocysts [23]; they have also been used on other invertebrates, such as the snail *Lymnaea stagnalis* [43], [44]. These antibodies recognize PKCα, β_I_, β_II_, δ, ε, η, and θ isotypes only when phosphorylated at a residue homologous to Ser660 of human PKCβ_II_ (Figure 1) that is crucial to activation [45]. Anti-phospho PKC (pan) (β_II_ Ser660) antibodies detected up to three immunoreactive bands in homogenates of adult *S. mansoni* (Figure 2B). Two faint bands were observed at ∼78 kDa and ∼132 kDa, similar to with anti-phospho PKC (pan) (ζ Thr410) antibodies; in addition, a more immunoreactive band of ∼116 kDa was seen, occasionally also detected with anti-phospho PKC (pan) (ζ Thr410) antibodies. This ∼116 kDa protein was only detected in cercariae and adult worms, with greater immunoreactivity in worm pairs (Figure 2B). No immunoreactive proteins were detected in sporocyst homogenates with anti-phospho PKC (pan) (Ser660) antibodies, and in miracidia only one protein was detected (∼78 kDa) (Figure 2B). This finding is in accord with our previously published work that demonstrated that this PKC became inactive during the miracidium-to-mother-sporocyst transition [23].

Finally, anti-phospho p44/42 MAPK (ERK1/2) (Thr202/Tyr204) antibodies have been used extensively to detect ERK phosphorylation (activation) in invertebrates including flies, snails and nematodes [44], [46]–[49], but not in *S. mansoni*. These antibodies detect ERK1/2 when phosphorylated at residues homologous to Thr202/Tyr204 of human ERK1 (Figure 1) with phosphorylation critical to activation. Three proteins were detected using these antibodies across the four life stages of *S. mansoni* investigated (Figure 2C). Proteins of ∼48 kDa and ∼43 kDa were detected in adult worm homogenates, with greater immunoreactivity consistently observed in males. In cercariae and sporocysts, a ∼43 kDa band and a weaker ∼38 kDa band were evident but the ∼48 kDa protein was not seen. The ∼43 kDa band was more intense in sporocysts than in cercariae, female worms or adult worm pairs (Figure 2C). Strikingly, there were no visible immunoreactive ERKs in miracidia homogenates (Figure 2C), when probing equal protein amounts from each life stage.

Treatment of blots containing protein extracts (20 µg) of adult worm pairs with lambda phosphatase for 4 h prior to exposure to each of the anti-phospho antibodies resulted in either a total loss (Figures 2B, 2C) or substantial reduction (Figure 2A) in immunoreactivity, demonstrating that the antibodies react specifically with the phosphorylated forms of these proteins.

### Immunodetected ERK- and PKC-like proteins from *S. mansoni* have characteristic ERK and PKC activities

**Figure 3:**
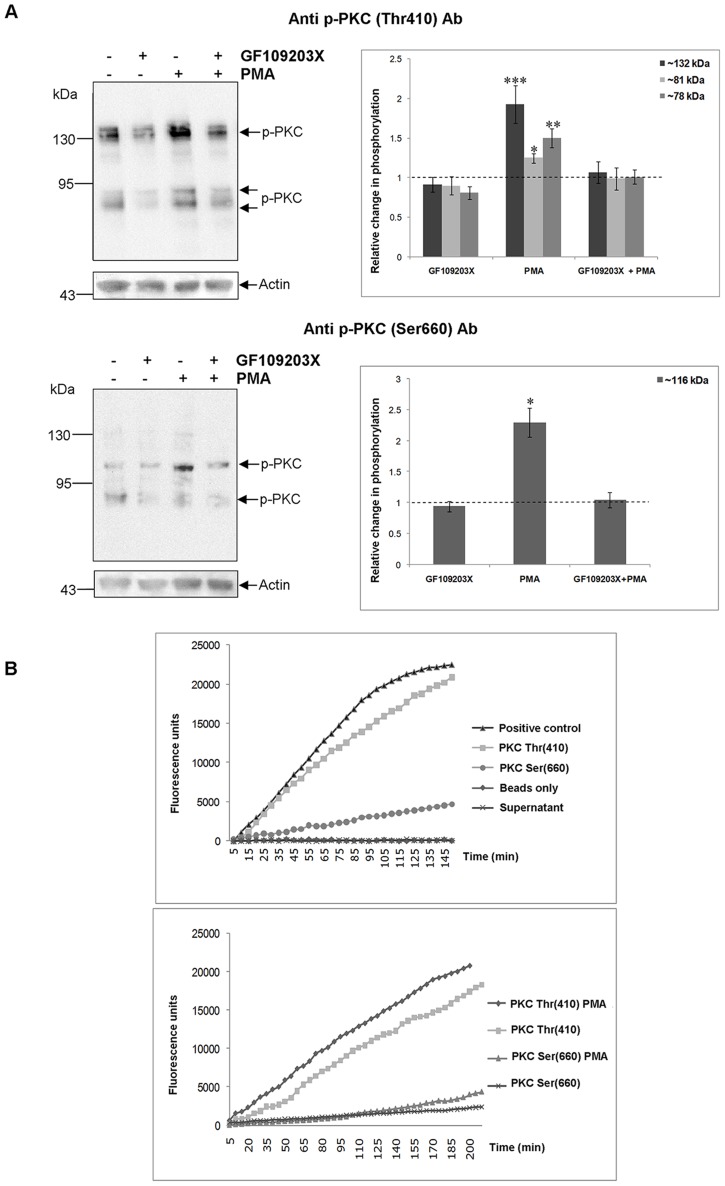
*S. mansoni* phosphorylated (activated) PKC-like proteins possess characteristic PKC activity. (A) Immunodetection of phosphorylated (activated) *S. mansoni* PKC-like proteins after exposure of live adult worm pairs to GF109203X (20 µM; 2 h), GF109203X (20 µM; 2 h) followed by PMA (1 µM; 30 min), or PMA (1 µM; 30 min) only. Protein homogenates (12 µg) were processed for Western blotting and blots probed with anti-phospho PKC (pan) (ζ Thr410) or anti-phospho PKC (pan) (βII Ser660) antibodies (Ab); anti-actin antibodies were used to assess protein loading between samples. Immunoreactive bands from three independent experiments, each with two replicates, were analyzed with GeneTools and the mean relative change (± SEM; see graphs) in phosphorylation for each protein calculated relative to the phosphorylation levels of untreated (DMSO or RPMI 1640) controls that were assigned a value of 1 (shown as the dotted line). * p≤0.05, ** p≤0.01, and *** p≤0.001 (ANOVA). (B) Adult *S. mansoni* were either left untreated (upper graph) or were exposed to PMA (1 µM) for 30 min or DMSO (vehicle) (lower graph) prior to homogenization; equal amounts of protein were then used for immunoprecipitation with anti-phospho PKC (pan) (ζ Thr410) or (βII Ser 660) antibodies, or for the negative (beads only) control. The PKC activities of immunocomplexes, negative control, supernatant, and positive control (recombinant human PKC) were then determined using the Onmia PKC assay kit with Ser/Thr 8 substrate peptide. Florescence was recorded every 5 min for up to 200 min. Results are representative of two independent experiments.

Next, we determined whether the phosphorylation (activation) state of the bands detected with the anti-phospho-PKC antibodies could be modulated using the PKC activator PMA or inhibitor GF109203X, reagents that we used previously to characterise PKC signalling in *S. mansoni* miracidia [23] and *L. stagnalis* haemocytes [49]. Phosphorylation of all bands increased significantly (p≤0.05) when live adult worms were exposed to 1 µM PMA for 30 min (Figure 3A), demonstrating that the endogenous PKC-like proteins are not fully activated in adults under physiological conditions. The ∼116 kDa and ∼132 kDa proteins showed the greatest increases in phosphorylation and are therefore likely cPKCs, which are characteristically highly responsive to DAG, Ca^2+^ and phorbol esters such as PMA [19]. A biphasic effect of PMA on PKC phosphorylation was also evident with increased phosphorylation observed between 15 and 30 min, followed by a decrease to basal levels at 60 min increasing again at 120 min (data not shown). GF109203X inhibits PKC activity *via* competition with the ATP binding site and does not directly inhibit active forms of the enzyme. Therefore, the inhibitory effect of GF109203X on *S. mansoni* PKCs was assessed by pre-incubating worms in GF109203X for 120 min, followed by exposure to 1 µM PMA for 30 min. Under these conditions, the PMA-induced phosphorylation of PKCs was blocked (Figure 3A). Then, to determine whether the immunoreactive phosphoproteins possessed PKC activity an immunoprecipitation/kinase assay was conducted. Adult worm proteins immunoprecipitated with either anti-phospho PKC antibody were able to phosphorylate the PKC substrate (Ser/Thr 8 peptide), with higher activity achieved with anti-phospho PKC (pan) (ζ Thr410) antibodies than with anti-phospho PKC (pan) (β_II_ Ser660) antibodies; activity also increased when worms were exposed to 1 µM PMA for 30 min (Figure 3B). The differences in kinase activity, seen as a result of the different antibodies having been used, is most likely due to the antibody affinity towards, and access to, the various epitopes in the native protein, together with the differential recognition of PKC isoforms displayed in Figure 3A.

**Figure 4:**
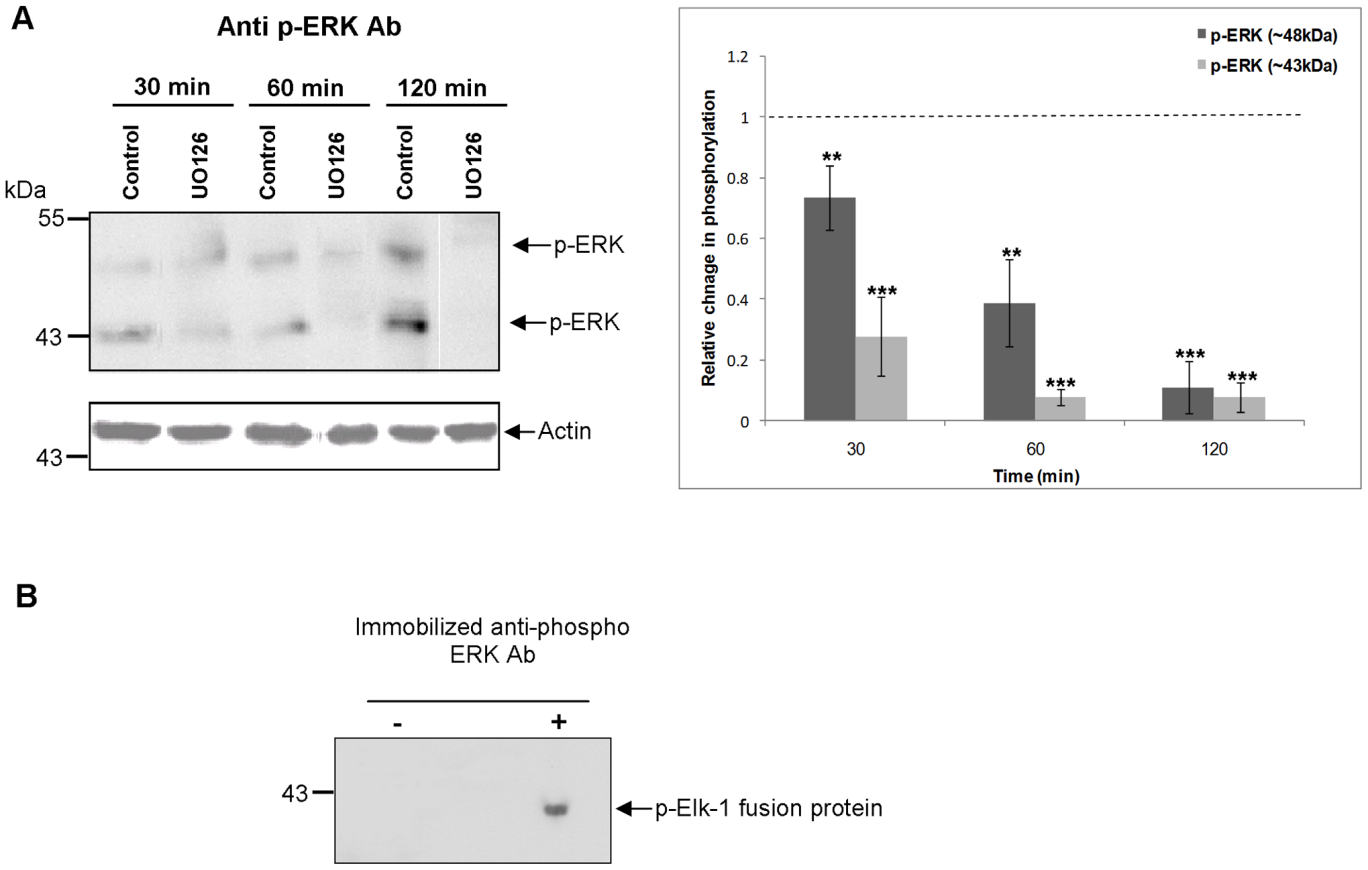
*S. mansoni* ERK-like proteins are phosphorylated (activated) by MEK and possess characteristic ERK activity. (A) Live adult worm pairs were exposed to 1 µM U0126 or 0.1% DMSO (control) for 30, 60 or 120 min. Protein homogenates (12 µg) were processed for Western blotting and blots probed with anti-phospho p44/42 MAPK (ERK1/2) (Thr202/Tyr204) antibodies (Ab). Immunoreactive bands from three independent experiments were analyzed with GeneTools and the mean relative change (± SEM; see graph) in phosphorylation of each protein calculated relative to the phosphorylation levels of controls that were assigned a value of 1 (shown here as the dotted line). **p≤0.01 and ***p≤0.001 (ANOVA). (B) Phosphorylation of Elk-1 fusion protein by immunoprecipitated ERK. Adult worm pairs were homogenized and equal amounts of protein used for immunoprecipitation with immobilized anti-phospho p44/42 MAPK (ERK1/2) (Thr202/Tyr204) antibodies or with beads only (negative control). Immunocomplexes were then incubated with Elk-1 fusion protein as the ERK substrate and phosphorylated Elk-1 was detected on Western blots using anti-phospho Elk-1 antibodies. Results are representative of two independent experiments.

Activated MEK1/2 is known to phosphorylate ERK1/2 in the TEY motif [30]. Therefore, to evaluate the effect of MEK inhibition on ERK phosphorylation, live adult worms were exposed to 1 µM of the MEK inhibitor U0126 for 30, 60 or 120 min. Immunoblotting revealed that U0126 significantly attenuated ERK phosphorylation over time, compared to controls (p≤0.05; Figure 4A). After 30 min, phosphorylation of the ∼48 kDa and ∼43 kDa proteins was attenuated by ∼27% and 72%, respectively (p≤0.01 and p≤0.001), and after 120 min ERK phosphorylation was almost completely blocked (Figure 4A). Furthermore, adult worm proteins immunoprecipitated with anti-phospho p44/42 MAPK (ERK1/2) (Thr202/Tyr204) antibodies phosphorylated the ERK substrate Elk-1 (Figure 4B), demonstrating that the detected ERK proteins display ERK activity.

Collectively these findings, when combined with knowledge of molecular weights (below) and antibody recognition sites, are entirely consistent with the detected proteins being *S. mansoni* PKCs and ERK1/2, with MEK acting upstream of ERK as in other organisms.

### The nature of the detected *S. mansoni* PKC and ERK proteins and their differential expression profiles

Having determined that the immunoreactive proteins behave like PKCs and ERKs, it was possible to consider these proteins in relation to the *S. mansoni* genome data sets. Although the precise assignment of Smp identifiers to immunoreactive bands was beyond the scope of this study, antibody recognition sites support a tentative assignment to the PKC class (Figure S3). As we have previously reported [23], the *S. mansoni* genome contains four annotated PKCs, two similar to human cPKCβI (Smp_128480, 75.6 kDa; Smp_176360, 114.9 kDa), one to nPKCε (Smp_131700, 94.9 kDa) and one to aPKCζ/ι (Smp_096310, 76.7 kDa), with PKCε also being similar to PKCη [4]. Of these molecules, the ∼78 kDa and ∼116 kDa phosphorylated PKCs detected by anti-phospho PKC (Ser 660) antibodies are most likely the β-type cPKCs (Smp_128480 and Smp_176360), because the crucial Ser residue recognized by these antibodies is conserved in these Smps and because the remaining *S. mansoni* nPKCε/aPKCι proteins do not possess this Ser autophosphorylation site (Figure 1). Moreover, the ∼81 kDa band detected exclusively with anti-phospho PKC (pan) (ζ Thr410) antibodies most likely corresponds to the atypical ι type PKC (Smp_096310) (Figure S3) as the Thr phosphorylation site (Thr489) is conserved but, as with human PKCι, the Ser phosphorylation site is not (Figure 1). The lack of strong activation of this PKC by PMA (Figure 3A) further supports this finding, given that aPKCs do not respond directly to DAG/PMA. Available transcriptomic data for cercariae, schistosomules and adult worms [50] (www.genedb.org) also confirm that these PKCs are expressed in these life stages, with expression being developmentally regulated (cf. Figure S3). The large ∼132 kDa PKC-like protein consistently detected in adult worms and cercariae using anti-phospho PKC (pan) (ζ Thr410) antibodies was activated by PMA and inhibited by GF109203X, confirming its PKC-like nature. Although absent from mammals, such high molecular weight PKCs are common in “lower” animals and, using similar antibodies, have been detected in the sea urchin *Lytechnus pictus* (135–140 kDa) [51] and *Caenorhabditis elegans* (122 kDa) [52]. PKC activation profiles were notably different among the four life stages studied, with four, two, and one PKC detected consistently in cercariae and adult worms, miracidia and mother sporocysts, respectively. This information suggests more complex roles for activated PKCs in the human host-infective/-dependent life stages. Wiest et al. [21] found nine-fold greater total PKC activity in adult *S. mansoni* than in larval stages, and it is established that PKC expression is developmentally regulated in other invertebrates [53]. We have shown that the ∼78 kDa β-type PKC of miracidia becomes inactive during development to the asexually reproducing, parasitic mother sporocyst stage and that PKC activity restricts this transformation [23]. Here, using anti-phospho (pan) (ζ Thr410) antibodies, we see that the ∼81 kDa PKC is substantially activated in 48 h mother sporocysts compared to miracidia, highlighting a possible role for this PKC in asexual reproduction and signifying the dynamic nature of PKC activation during schistosome development.

The ∼48 kDa and ∼43 kDa proteins detected with anti-phospho p44/42 MAPK antibodies likely correlate to genome sequences Smp_142050 (45.3 kDa, 70% identity to human ERK1) and Smp_047900 (SmERK2, 40.83 kDa, 68% identity to human ERK1), due to molecular weight and antibody detection site similarity (Figures 1, 2C and S3); these proteins are also expressed in the requisite life-stages [50] (www.genedb.org) (Figure S3). The recognition site of the p44/42 MAPK antibodies was conserved among three other putative *S. mansoni* ERK-like proteins (Smp_133490, 58.7 kDa; Smp_133500, 82.7 kDa; Smp_134260, 70 kDa), however, phosphorylated ERK-like proteins of this size were not detected on blots. The ∼38 kDa protein detected in mother sporocysts and weakly in cercariae is possibly a non-specific band, because ∼38 kDa ERK-like proteins could not be detected in the *S. mansoni* genome. The ERKs also appear differentially activated in the different *S. mansoni* life stages studied (Figure 2C), with ERK1/2 (∼48/∼43 kDa) detected only in adult worms. No ERK activation was detected in miracidia, and only the ∼43 kDa ERK was phosphorylated (using 12 µg protein) in mother sporocysts and cercariae. Thus, the ∼48 kDa ERK in male worms may play a specific role in growth and development and/or host-interactions, particularly given that human EGF activates EGFR in *S. mansoni* [33] and influences ERK signalling and proliferation in other parasites, including *Echinococcus multilocularis* [54] and *Trypanosoma cruzi* [55]. The ERK pathway also interacts with the transforming growth factor β (TGFβ) pathway in schistosomes, possibly restricting interaction of SmSmad4 with receptor-activated Smad2 [56]. Therefore, because TGFβ signalling plays a part in mitotic activity, parasite development and egg embryogenesis [57], [58], ERK activity in particular cell types might suppress TGFβ signalling, with concomitant effects on development and reproduction.

### Connectivity between *S. mansoni* ERK and PKC signalling

**Figure 5:**
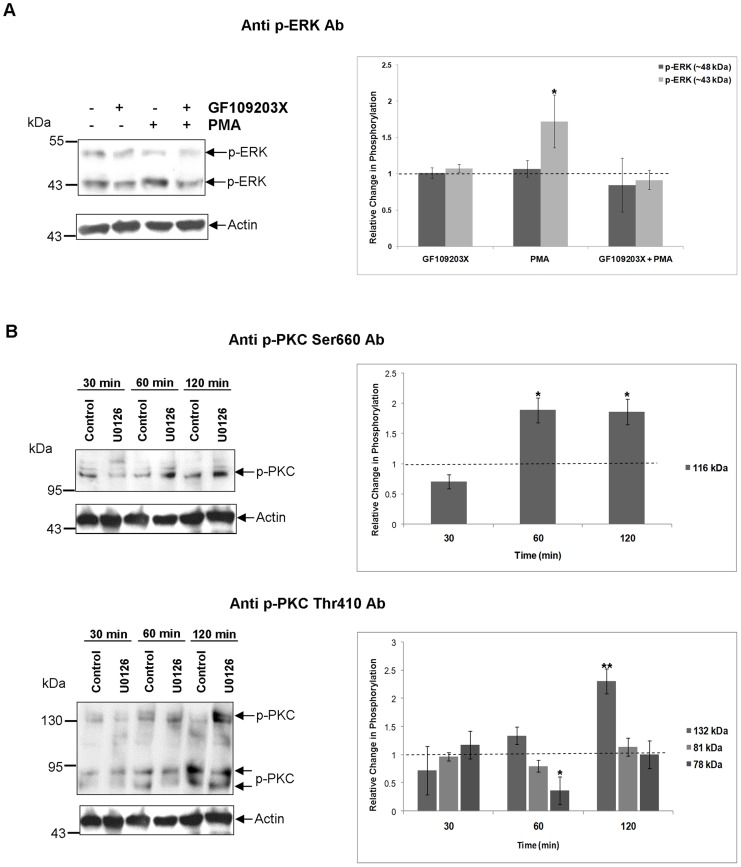
Connectivity between ERK and PKC signalling in *S. mansoni*. Adult *S. mansoni* were treated with (A) GF109203X (20vM; 120 min), PMA (1 µM; 30 min), GF109203X (20 µM; 120 min) followed by PMA (1 µM; 30 min), or DMSO (vehicle control), or with (B) U0126 (1 µM; 30, 60, and 120 min) or DMSO (vehicle control). Proteins (12 µg) were then processed for Western blotting using (A) anti-phospho p44/p42 MAPK (ERK1/2) (Thr202/Tyr204), or (B) anti-phospho PKC (pan) (ζ Thr410) or anti-phospho PKC (pan) (βII Ser660) antibodies (Ab). Anti-actin antibodies were used to assess protein loading. Immunoreactive bands from three independent experiments were analysed and quantified using GeneTools and the mean relative change (± SEM; see graphs) in phosphorylation for each protein calculated relative to the phosphorylation levels of untreated (DMSO or RPMI-1640) controls that were assigned a value of 1 (shown as the dotted line). *p≤0.05, **p≤0.01 (ANOVA).

Cross talk between PKC and ERK signalling, with PKC upstream of ERK is common in many organisms, including invertebrates such as *C. elegans* [59]. To determine whether ERK and PKC signalling are connected in *S. mansoni*, ERK phosphorylation was determined after live adult worms were exposed to PKC modulators, and *vice-versa*. PMA (1 µM) increased phosphorylation of the ∼43 kDa ERK (p≤0.05; 1.8 fold) after 30 min, however, the ∼48 kDa ERK was unaffected (Figure 5A). This activation was blocked by treatment with GF109203X for 120 min prior to PMA stimulation, supporting ERK's dependency on PKC in response to PMA (Figure 5A). Treatment with GF109203X alone did not inhibit the phosphorylation of either ERK after 120 min incubation (Figure 5A), likely because this inhibitor only prevents activation of inactive PKC and because ERK may also be activated independently of PKC. On the other hand, exposure of worms to 1 µM U0126 for 60 or 120 min stimulated phosphorylation of the ∼116 kDa PKC (p≤0.05; ∼1.9-fold), and of the ∼132 kDa PKC after 120 min (p≤0.01; ∼2.5-fold) (Figure 5B). Interestingly, phosphorylation of the 78 kDa PKC was suppressed after 60 min U0126 treatment but recovered after 120 min (Figure 5B). Collectively, these findings imply that PKC lies upstream of ERK in *S. mansoni* and suggest that complex temporally regulated feedback to PKC can occur in response to MEK inhibition.

### Activated PKC and ERK in *S. mansoni* adult worms is associated with muscular, tegumental and reproductive structures

**Figure 6:**
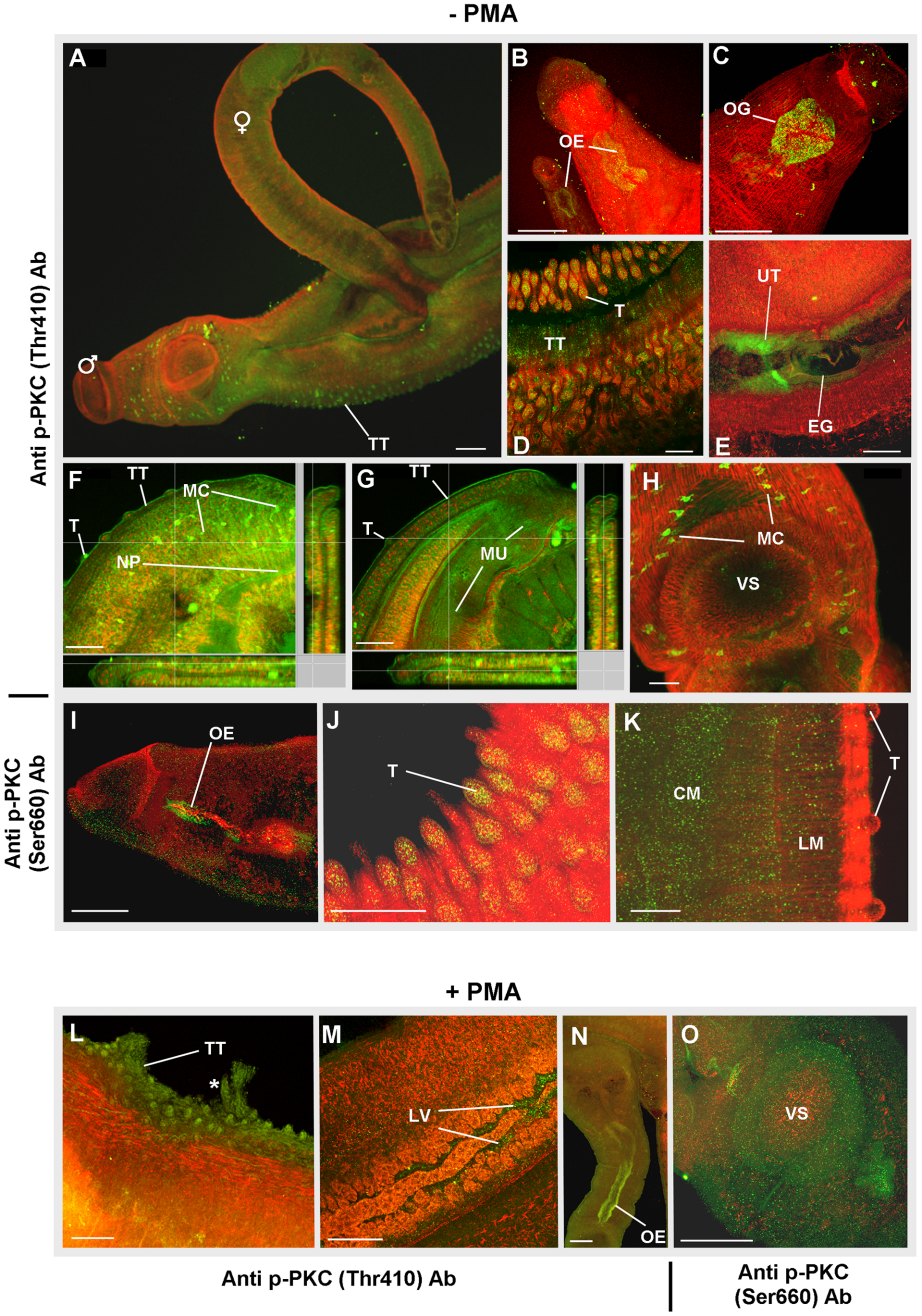
Immunolocalization of phosphorylated (activated) PKC in intact *S. mansoni*. Adult worm pairs were either (A–K) fixed immediately after perfusion, or (L–O) treated with PMA (1 µM; 15 min) before fixing. Worm pairs were then incubated with anti-phospho PKC (pan) (ζ Thr410) or anti-phospho PKC (pan) (βII Ser660) primary antibodies (Ab) and Alexa Fluor 488 secondary antibodies (green); specimens were additionally stained with rhodamine phalloidin to reveal actin filaments (red). Activated PKC was found associated with: (B, C, I, N) oesophagus (OE) and oesophageal gland (OG); (A, D, F, G, J–L) tubercles (T) of the tegument (TT); (E, M) reproductive structures including the uterus (UT) surrounding an egg (EG) during expulsion and lumen of the vitellaria (LV); (K) longitudinal (LM) and circular muscle (CM) layers; (F,G) myocytons (MC) including those close to the (H) ventral sucker (VS); (O) the VS; and (F,G) the neural plexus (NP) innervating the musculature (MU). (L) The asterisk (*) identifies an area of the tegument possibly disrupted by PMA. All images are of z-axis projections displayed in maximum pixel brightness mode. Bar: A = 100 µM, B–O = 50 µm.

Identifying where cell signalling pathways in *S. mansoni* are predominantly activated is valuable in helping to unravel their function. Therefore, we performed functional mapping of phosphorylated PKC and ERK in freshly perfused, paired, intact *S. mansoni* adults using our recently published approaches [60]. We preferred this approach to *in situ* hybridization with gene specific probes, because we were interested in localizing functionally active kinases rather than protein expression *per se*. In all cases, negative control worms displayed minimal background staining (Figure S1). Staining with anti-phospho PKC (pan) (ζ Thr410) antibodies revealed activated PKC associated with the tegument and tubercles of the tegument (Figures 6A, 6D, 6F and 6G), particularly in the central dorsal-lateral region of males where tubercles are most prominent. In addition, activated PKC was associated with the oesophagus (Figure 6B), oesophageal glands (Figure 6C), and the uterus of the female in the vicinity of eggs (Figure 6E). In males, deeper confocal scanning revealed PKC activation in the muscle layers, neural plexus, and myocytons (Figures 6F–H). Worms labelled with anti-phospho PKC (pan) (β Ser660) antibodies displayed generally weaker fluorescence; however, areas with notable PKC activation included the oesophagus (Figure 6I), tubercles on the dorsal side of the male (Figure 6J), and muscle layers under the tegument (Figure 6K). Live adult worms were also exposed to 1 µM PMA for 30 min in an attempt to identify other regions displaying PKC activation. In these specimens, activation was evident in similar regions to those observed without PMA treatment; however, generally more activation was observed at the tegument surface (Figure 6L), and evidence of tegument disruption was apparent in some specimens in response to PMA, as has been reported by other authors [61]. Activated PKC was also found to be associated with the lumen of the vitellaria, more extensive portions of the oesophagus and the muscular ventral sucker (Figures 6M–O). Although, in a recent study, the transcription of the β-type PKC-encoding gene (Smp_176360) was found to be upregulated ∼3.2 times in the testes of adult males compared to whole worms [62], no significant PKC activation was seen in this tissue in the current study.

**Figure 7:**
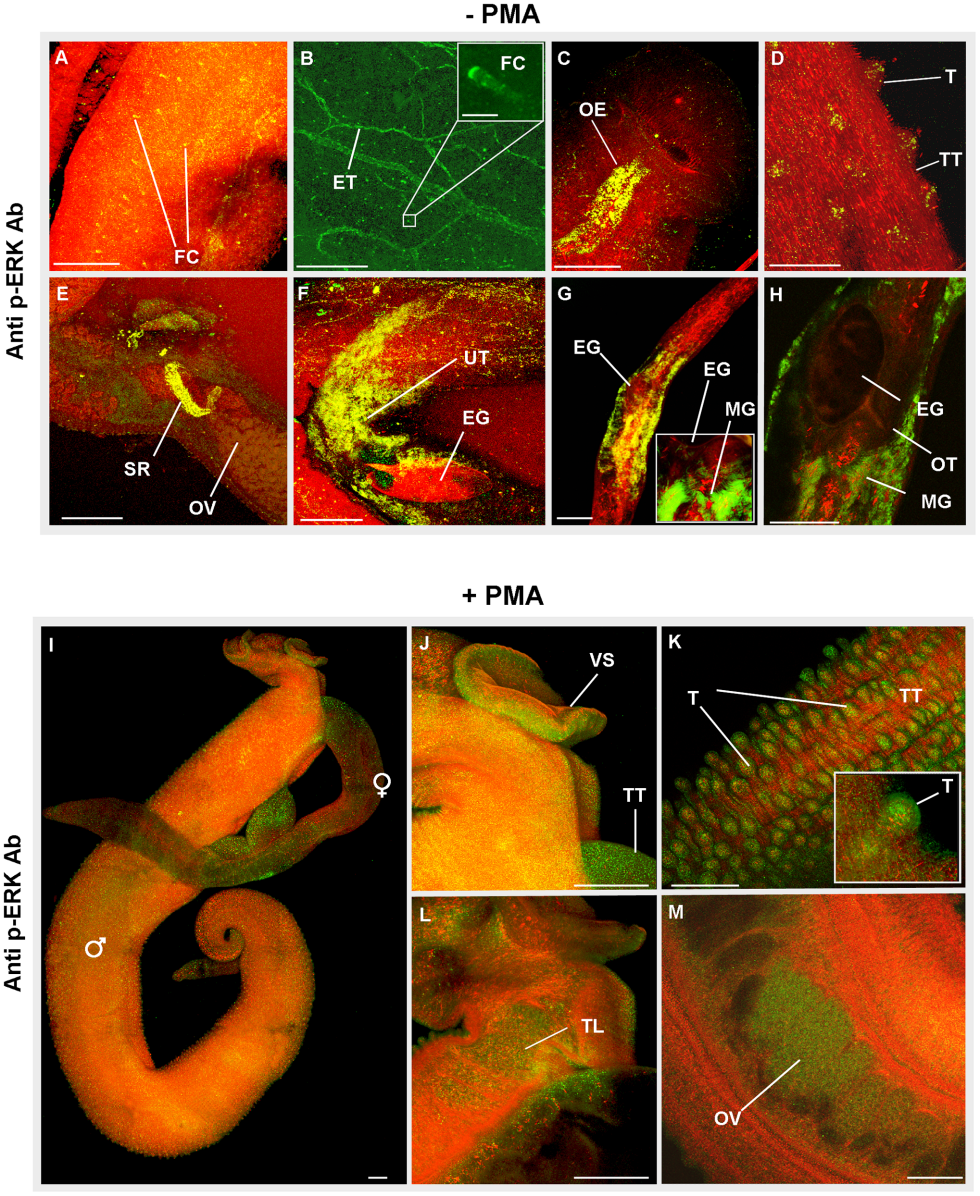
Immunolocalization of phosphorylated (activated) ERK in intact *S. mansoni*. Adult worm pairs were either (A–H) fixed immediately after perfusion, or (I–M) treated with PMA (1 µM; 15 min) before fixing. Worm pairs were then incubated in anti-phospho p44/42 MAPK (ERK1/2) (Thr202/Tyr204) antibodies and Alexa Fluor 488 secondary antibodies (green); specimens were additionally stained with rhodamine phalloidin to reveal actin filaments (red). Under normal conditions activated ERK was found associated with: (A, B) flame cells (FC) and excretory tubules (ET); (C) oesophagus (OE); (D) tubercles (T) of the tegument (TT); (E) seminal receptacle (SR) adjacent to the ovary (OV); (F) the uterus (UT) surrounding an egg (EG) during expulsion; (G, H) the region of the Mehlis' gland (MG) adjacent to the ootype (OT) containing the egg. Following exposure to PMA increased ERK activation was evident over (I) the worm surface including at the (J) ventral sucker (VS), and (K) the tubercles (T) of the tegument (TT). Activated ERK was also associated with (L) the testicular lobes (TL) and (M) the ovary (OV). All images are of z-axis projections displayed in maximum pixel brightness mode. Bar:  = 50 µm.

In untreated male worms, ERK activation was particularly evident in excretory tubules and flame cells (Figure 7A, 7B), the oesophagus (Figure 7C) and tubercles of the tegument (Figure 7D). In females, regions displaying notable ERK activation included the seminal receptacle (Figure 7E), uterus surrounding the egg during expulsion (Figure 7F), ootype wall and the region of Mehlis' gland (Figures 7G–H). Upon exposure to 1 µM PMA for 30 min, activated ERK was observed in regions similar to those of untreated samples; however, stronger immunoreactivity was apparent in the tegument and tubercles (Figures 7I–K). Additionally, activated ERK was evident in the male ventral sucker (Figures 7I–J), the testicular lobes (Figure 7L), and ovary (Figure 7M), possibly due to the ∼43 kDa ERK, as only this isotype responded to PMA (Figure 5A). Interestingly, ERK1 (Smp_142050) expression was recently found to be ∼2.7 and ∼3.9 times higher in the ovary and testes of adult female and male worms, respectively, when compared to whole worms [62]. Activated ERK has also been reported in the tegument of the cestode *T. crassiceps* [63], where it is thought to interact with host factors.

That PKC and ERK reside within the tubercles and tegument of *S. mansoni* and that cross talk many occur between these pathways is exciting, because the tegument of schistosomes is in constant contact with host blood. The tegument is a dynamic structure, where considerable host interplay is expected to take place [64]. EGFRs [65] and GPCRs [66] localize to the tegument and could activate PKCs and ERKs, possibly in response to host growth factors/hormones, which might influence schistosome development/survival. Localization of activated PKC to the musculature, neural plexus and myocytons supports a role for PKC in motor activity. Recently, we reported that activated protein kinase A (PKA) localizes to the central and peripheral nervous system of *S. mansoni* and that PKA activation results in hyperkinesia, demonstrating a role for PKA in neuromuscular co-ordination [60]. Activated PKA also localizes to the tegument, tubercles and canyons between the tubercles [60]. This finding, coupled with results described here, supports that complex signalling occurs at the schistosome surface. Finally, the localization of activated PKC/ERK to the oesophageal glands/oesophagus, uterus during egg expulsion, the ootype, Mehlis' gland, seminal receptacle and the excretory system highlights the likely importance of these kinases to schistosome reproduction and homeostasis. Increased pumping of the pharynx/oesophagus muscle during *C. elegans* starvation is dependent on ERK activity through PKC phosphorylation [67]. We hypothesize, therefore, that PKC/ERK regulate pumping of the schistosome oesophagus and that interference of these pathways may restrict blood feeding.

### Pharmacological modulation of PKC and ERK activity induces physiological disturbance in adult *S. mansoni*

To discover physiological roles for PKC and ERK in *S. mansoni* the effects of kinase modulation on adult worm phenotype were evaluated over 96 h. A range of inhibitor concentrations was used with doses chosen to reflect that whole, large, worms were exposed to each inhibitor rather than single cells. Five parameters were quantified: separation of worm pairs, male ventral sucker detachment (in paired and unpaired males), egg output (per couple), speed of movement (velocity, mm/s) of the worm posterior tip (a proxy for muscular activity), and persistent coiling. In RPMI 1640, worms remained paired releasing 103–110 eggs/day/couple during the first 72 h, decreasing to 93 thereafter (data not shown). Males remained attached to culture plates during the first 24 h and only 5–7% detached between 24 h and 96 h. The worm posterior tip velocity of couples was 3.26 mm/s (+/−0.33 mm/s) after 1 h increasing to 3.68 mm/s (<+/−0.55 mm/s) thereafter. Extensive and/or sustained coiling was seldom observed. There were no significant differences between DMSO and RPMI 1640 control groups for these parameters. Worms displayed normal physiology (Movie S1).

**Figure 8:**
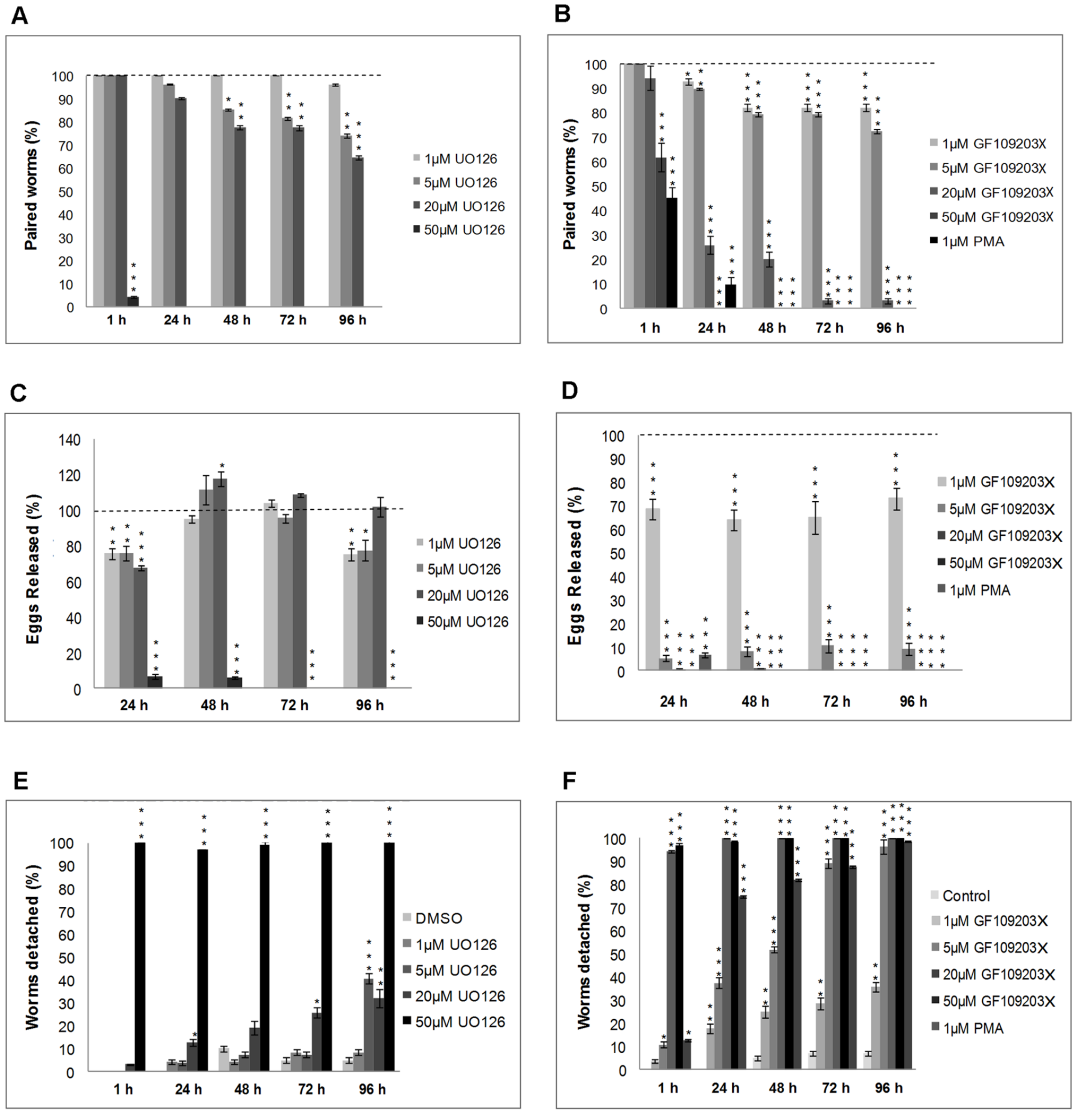
Modulation of PKC and ERK activity in adult *S. mansoni* induces worm uncoupling, suppresses egg output, and causes male worms to detach. Adult worm pairs were incubated in increasing concentrations of U0126, GF109203X, or 1 µM PMA and movies captured at various time points over 96 h and imported into Image J. The effects of these compounds on (A, B) pairing, (C, D) egg release by paired worms (uncoupled worms were not included), and (E, F) male adult worm detachment from the culture plate were then determined against vehicle controls (which in A–D were assigned a value of 100% and are shown as the dotted line). Mean values (± SEM) shown represent those from four independent experiments, each of which contained a minimum of six adult worm pairs. *p≤0.05, **p≤0.01, and ***p≤0.001 (ANOVA).

**Figure 9:**
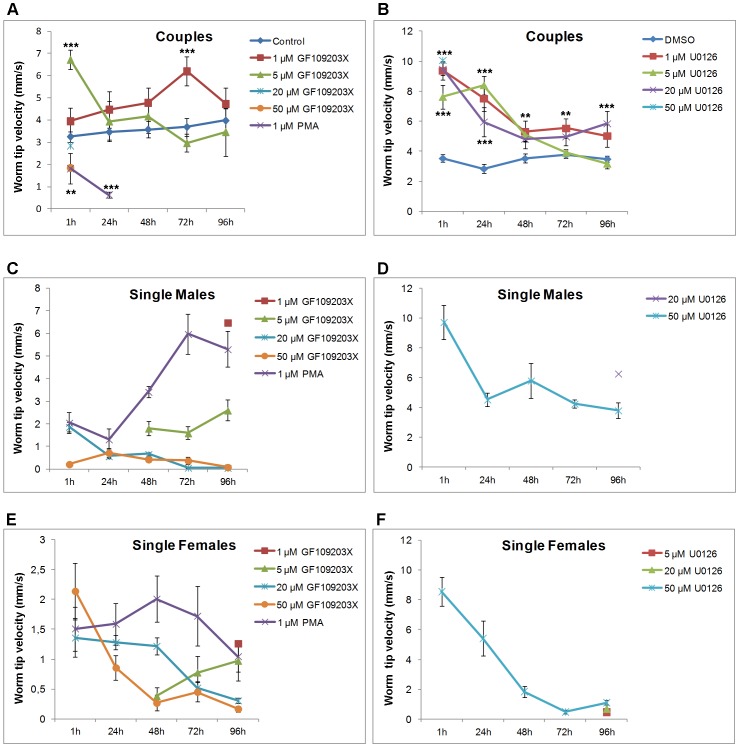
Modulation of PKC and ERK activity in adult *S. mansoni* affects worm movement. Adult worm pairs were incubated with increasing concentrations of U0126, GF109203X, or 1 µM PMA and movies captured at various time points over 96 h and imported into Image J. Movies were then analyzed to determine the speed of movement (velocity, mm/s) that the posterior tip of each worm travelled in 10 s when (A, B) coupled. In some cases worms uncoupled as a consequence of treatment (as shown in Figure 8A, B). When this was the case, the speed of movement of the uncoupled (C, D) single male or (E, F) female worms was determined for their respective treatments for the remainder of the assay; note that no vehicle control exists when worms had separated because the control worms remained paired. Mean values (± SEM) shown represent those from four independent experiments each of which contained a minimum of six adult worm pairs; the actual numbers of worms in each analysis is, however, dependent upon the uncoupling effect of the individual treatment (shown in Figures 8A, B). *p≤0.05, **p≤0.01, and ***p≤0.001 (ANOVA).

In contrast, U0126, which was shown to attenuate ERK activation, increased worm unpairing with 50 µM causing 96% separation within 1 h (p≤0.001) (Figure 8A; Movies S2 and S3). This coincided with rapid detachment of males from culture plates (p≤0.001) (Figure 8E, Movie S3). Lower U0126 concentrations had more subtle but still significant effect; after 96 h, 26% (p≤0.01) and 35.5% (p≤0.001) of adult worms separated, and 44.7% (p≤0.001) and 29% (p≤0.01) of males detached from plates with 5 µM and 20 µM U0126, respectively (Figures 8A, 8E). With 20 µM U0126 egg output declined at 24 h but increased between 24 and 48 h (p≤0.01) (Figure 8C). This early suppression of oviposition is interesting given that activated ERK was observed in the seminal receptacle, uterus, ootype region and Mehlis' gland. Egg production essentially ceased at 50 µM U0126 with only a few (2–3) small immature eggs sporadically found, possibly a consequence of worm separation (Figure 8C). U0126 also affected worm motion. At 1 h, 1, 5, 20 or 50 µM U0126 increased tip velocity (when worms were paired) by 265% (to 9.37 mm/s; p≤0.001), 203% (to 7.62 mm/s; p≤0.01), 251% (to 8.87 mm/s; p≤0.001) and 285% (to 10.66 mm/s (p≤0.001), respectively, compared to DMSO control speeds of 3.54 mm/s. However, movement decreased temporally and at 96 h velocities were between 3.17 and 5.85 mm/s (Figure 9B). Because worms separated rapidly in 50 µM U0126, tip velocities for the resultant unpaired worms were also determined for the remainder of the assay while in the inhibitor. These single males and females displayed high posterior tip velocities after 1 h with speeds of 9.74 and 8.5 mm/s, respectively (Figure 9D, 9F); thereafter, movement decreased and at 96 h males were 50% and females 90% slower. An uncoordinated ‘jerky’ behavior was observed at all U0126 doses (Movie S3).

**Figure 10:**
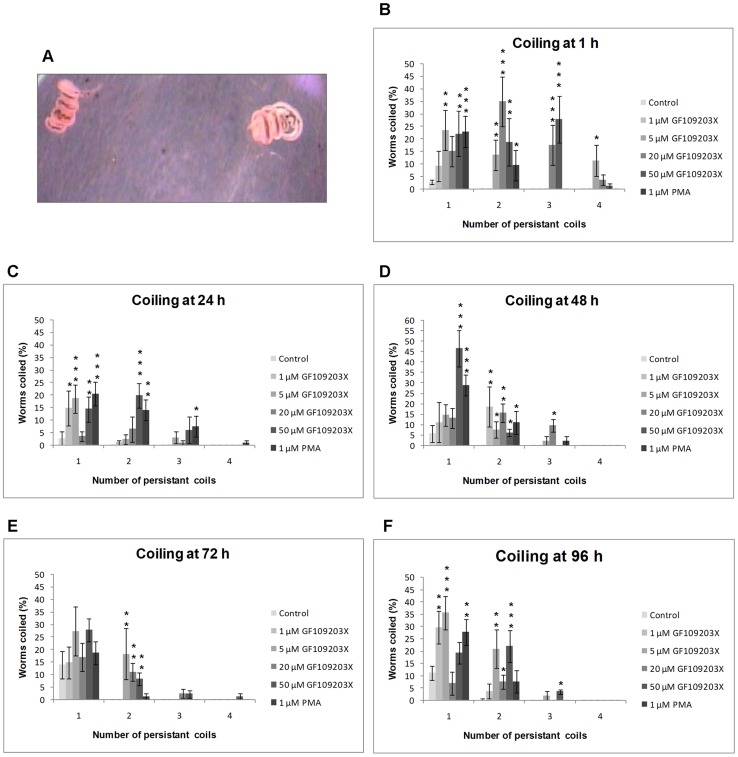
Modulation of PKC activity in adult *S. mansoni* induces sustained coiling. Adult worm pairs were incubated with increasing concentrations of GF109203X, or 1 µM PMA and movies captured at (B) 1 h, (C) 24 h, (D) 48 h, (E) 72 h, and (F) 96 h and imported into Image J. Movies were then analyzed to determine the number of coils (shown in A) present per worm that were sustained for at least 10 s. Values shown represent the mean (± SEM) percentage of worms displaying a given number of coils at each time point for each treatment. *p≤0.05, **p≤0.01, and ***p≤0.001 (ANOVA).

GF109203X inhibited worm pairing, egg output, and ventral sucker attachment (Figure 8B, 8D, 8F) and also induced extensive and persistent worm coiling (Figure 10; Movie S4). During the first hour, a proportion of worms remained paired at all GF109203X concentrations, and at >1 µM pairs coiled persistently which seemed to be caused by males, sometimes resulting in expulsion of female gut contents (Movies S4 and S5). After 1 h in 20 µM GF109203X, 94% of males had detached from plates (p≤0.001) (Figure 8F). Between 24 h and 96 h all worms separated in 50 µM GF109203X, with separation also observed at lower concentrations (p≤0.001) (Figure 8B). Egg output was also completely blocked within 24 h (Figure 8D) with 20 µM or 50 µM GF109203X (p≤0.001) (Figure 8D). This reduction in egg output was sustained over 96 h despite couples remaining. This could be due to PKC inhibition affecting uterine muscular movement as proposed for PKA [60], or to direct effects on egg fertilization/formation. In the context of coiling, although worms displaying three coils or more were most prevalent during the initial 48 h GF109203X treatment (Figure 10) worms had an increased propensity to coil throughout the experiment. For instance after 1 h in 5, 20 or 50 µM GF109203X only 52%, 26% and 30% worms exhibited their normal shape, respectively (p≤0.001), with the remainder possessing one or more sustained coils (Figure 10B). Worm tip velocity of couples was inhibited by 50 µM GF109203X only, to 1.9 mm/s (p≤0,001) or 60% that of controls (Figure 9A). However after worms separated in response to GF109203X movement reduced, with striking effects seen at 96 h (p≤0,001) when the single males displayed velocities of just 0.06 mm/s and 0.09 mm/s, and the single females 0.3 mm/s and 0.17 mm/s for 20 µM and 50 µM GF109203X, respectively (Figures 9C, 9E; Movie S4).

PMA (1 µM) also induced rapid worm separation and only 10% remained paired at 24 h (p≤0.001) and none at 48 h (Figure 8B; Movie S6). At least 75% of worms detached from plates after 24 h rising to over 97% after 96 h. Initially, the separated males showed reduced movement in PMA and separated female velocities were similar to those of remaining control pairs, however, over time the male worm tip velocities increased to 5.3 mm/s (Figure 9C). No eggs were produced in PMA by worms (Figure 8D), persistent coiling was absent and worms appeared rigid, although this became less prominent over time (data not shown).

Blair et al. [20] first alluded to a role for PKC in *S. mansoni* muscle contraction. Our detailed analyses substantiate this role, and link aberrant phenotypes induced by disrupted signalling to anatomical findings for activated PKC (and ERK) identified using functional kinase mapping. Extensive labelling of activated PKC in the muscle with activation also apparent in the neural plexus and myocytons is in accordance with effects of PKC modulation on worm motility, coiling and male ventral sucker detachment. PKCs, particularly PKCβ, mediate smooth muscle contraction in vertebrates [68], [69], and, in *C. elegans*, PKCα/PKCβ-like PKC2 isotypes are expressed in neurons and muscle cells [40]. In addition, PKC inhibitors block FMRF-amide induced muscle contraction in *S. mansoni* [70]. Despite having opposing effects on PKC phosphorylation, PMA and the higher doses of GF1090203X affected ventral sucker attachment and worm pairing similarly over 96 h (Figures 8B, 8D, 8F) and tip velocity within the initial 24 h (Figures 9A, 9C, 9E); however, PMA did not induce coiling. Although PMA activates PKC, overnight phorbol ester treatment is used to down-regulate cPKC expression in mammalian cells [71]. Therefore, we exposed live adult worm pairs to PMA (1 µM) for 24 h. Western blotting with anti-phospho PKC (pan) (ζ Thr410) and anti-phospho PKC (pan) (βII Ser660) antibodies revealed that such treatment suppressed activation of PKCs to between 80% (±0.06%; p≤0.001, n = 3; for the ∼132 KDa PKC) and 64% (±0.1%; p≤0.05, n = −3; 116 kDa PKC) (Figure S2). Although we could not ascertain whether PKC protein expression was reduced, because we lacked an antibody that satisfactorily detects total PKC protein levels, the significant reduction in phosphorylation of treated worms demonstrates pathway deactivation, the critical parameter in terms of function. This information also suggests that decreased expression likely occurred. Therefore, the effects of PMA treatment on worm phenotype after 24 h might be expected to reflect those seen with GF109203X, which they do in part. However, because PMA causes tegumental disruption [61] stress-related effects on phenotype are possible. The role of ERK in schistosome muscle contraction requires further investigation. Whereas MEK inhibition increased motility of worm pairs, motility of single females reduced considerably over 72 h and was lower than that of single males (Figures 9B, 9D, 9F). Nevertheless, that U0126 affected worm motility, attachment and pairing, highlights the ERK pathway, together with the PKC pathway, as an important regulator of schistosome muscle homeostasis.

### Praziquantel induces activation of *S. mansoni* PKCs and ERKs

**Figure 11:**
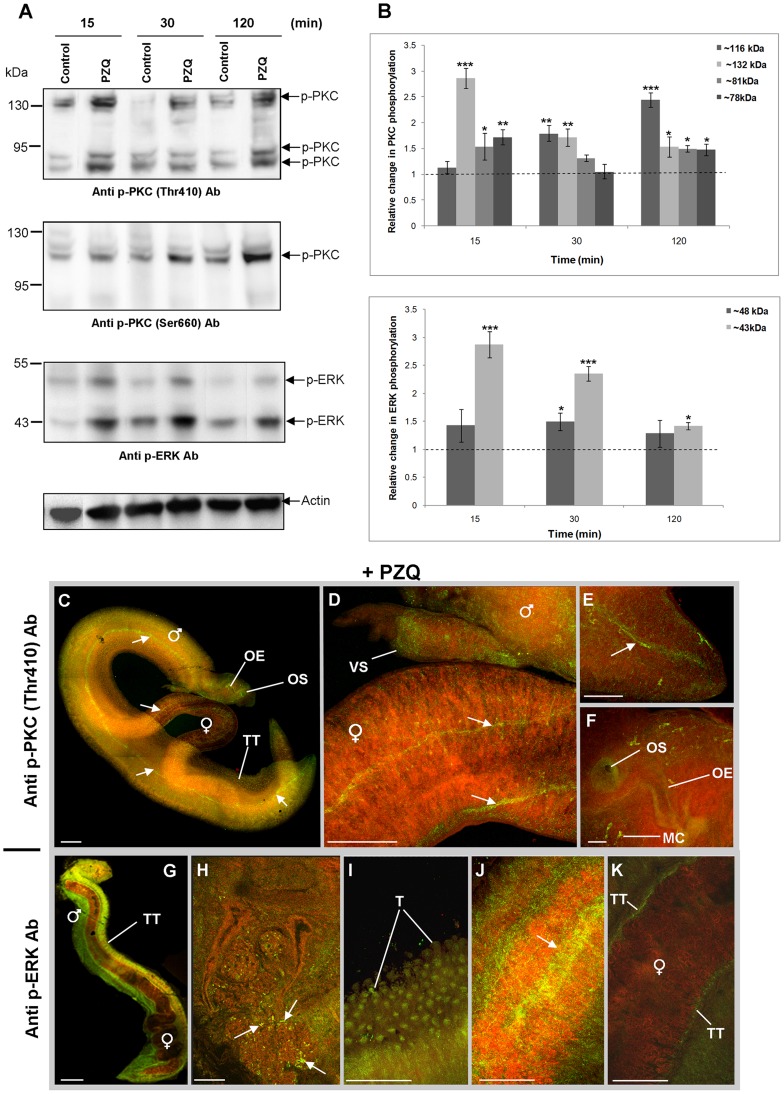
Praziquantel (PZQ) induces the phosphorylation (activation) of ERK and PKC in adult *S. mansoni*. (A) Live adult worms were exposed to 0.02 mg/ml PZQ or 0.2% DMSO (vehicle control) for 15, 30 and 120 min. Proteins (12 µg) were processed for Western blotting and blots probed with anti-phospho PKC (pan) (ζ Thr410), anti-phospho PKC (pan) (βII Ser660), or anti-phospho p44/42 MAPK (ERK1/2) (Thr202/Tyr204) antibodies (Ab); anti-actin antibodies were used to assess protein loading differences between samples. (B) Immunoreactive bands from three independent experiments, each with two replicates, were analyzed and mean relative change (± SEM) in phosphorylation for each protein calculated relative to the phosphorylation of controls that were assigned a value of 1 (shown as the dotted line). *p≤0.05, **p≤0.01, and ***p≤0.001 (ANOVA). (C–K) Adult worm pairs were treated with 0.02 mg/ml PZQ for 15 min prior to fixing. Worm pairs were then incubated in either (C–F) anti-phospho PKC (pan) (ζ Thr410) or (G–K) anti-phospho p44/42 MAPK (ERK1/2) (Thr202/Tyr204) primary antibodies, followed by Alexa Fluor 488 secondary antibodies (green); specimens were also stained with rhodamine phalloidin to show actin filaments (red). Activated PKC was found particularly associated with: (C) the tegument (TT) and oesophagus (OE); (C, F) opening of the oral sucker (OS) and myocytons (MC); (D) the musculature of the ventral sucker (VS); and (C–E) collecting ducts of the excretory system (arrowed). Activated ERK was found particularly associated with (G, I) tubercles (T) of the tegument (TT); (H) cephalic ganglia (arrowed); (J) musculature (arrow); and (K) surface of the female worm tegument (TT) adjacent to the male gynaecophoric canal. All microscopy images are of z-axis projections displayed in maximum pixel brightness mode Bar: C, D, G, K = 100 µm; E, F, H, I = 25 µm.

Because PZQ affects cellular calcium dynamics [72], [73] and cPKCs are calcium responsive [19] we hypothesized that PZQ would affect the activation of *S. mansoni* PKCs and ERKs. After 15 min PZQ treatment, worm pairs became contracted and immobile and assumed a shrunken appearance (data not shown); coincident with this, the ∼78, ∼81, and ∼132 kDa PKCs detected with anti-phospho PKC (pan) (ζ Thr410) antibodies showed increased phosphorylation (p≤0.05; Figures 11A, 11B). Although the ∼116 kDa PKC detected by anti-phospho PKC (pan) (βII Ser 660) antibodies was unaffected by PZQ at 15 min, increased phosphorylation was observed at 30 min and 120 min (p≤0.001; Figures 11A, 11B). The ∼43 kDa ERK was also activated in response to PZQ treatment displaying a 2.9-fold increase in phosphorylation at 15 min (p≤0.001; Figure 11A, 11B) whereas the ∼48 kDa ERK was less affected.

Confocal laser scanning microscopy revealed that PZQ-treated worms possessed strong ERK and PKC activation, with generally more signal in males than females (Figures 11C–K). Activated ERK was associated with the tegument of paired male and female worms, male sub-tegumental regions, musculature, and cephalic ganglia on the male head (Figures 11G–K). Anti-phospho PKC (pan) (ζ Thr410) antibodies revealed activated PKC associated with tegumental and subtegumental regions, the oral sucker and oesophagus and structures along the dorsal side of male and female worms resembling collecting ducts (Figures 11C–F) which were also faintly seen in some PMA treatments (data not shown). The pattern of activation revealed using anti-phospho PKC (pan) (βII Ser 660) antibodies was similar that in the absence of PZQ, however, low levels of PKC activation were also seen in the testicular lobes (data not shown).

Despite decades of use the precise action of PZQ remains unresolved. Adult worms exposed to PZQ become paralyzed, lose ion homeostasis and take up calcium [72]–[74]; tegument disruption ensues with an associated exposure of worm antigens at the surface [75]. Mature males are more sensitive towards PZQ than females; additionally juvenile 28-day old worms, which are refractory to PZQ, take up large quantities of calcium, implying that PZQ sensitivity is not entirely due to calcium influx [39]. Possible targets of PZQ include a Ca_v_β ion channel subunit [76], myosin light chain [77] and actin [78]. Sustained worm contraction typical of PZQ treatment was observed here when worms were exposed to PMA for 1 h (Figure 9A); thus, PZQs affect on worm motility is likely mediated through PKC and ERK. Interestingly, 28-day old worms exposed to PZQ for 20 h displayed a 6-fold increase in the gene coding for a suppressor of MEK [79]; this could serve to dampen PZQ-dependent ERK activation. Moreover, recently, PKC expression was found to be upregulated in adult male and female *S. japonicum* when exposed *in vivo* to a sub-lethal PZQ dose for 12 h or more, possibly in response to the increased calcium influx [80]. Therefore, whether the activation of PKC and/or ERK influences the outcome of worm survival in the face of PZQ treatment should be further investigated.

### Conclusions and context for schistosome control

To conclude, we have, for the first time, functionally characterised global signalling by PKCs and ERKs in adult *S. mansoni* and, using functional mapping, have localized PKC and ERK activities to numerous anatomical regions important to schistosome motor activity, excretion, reproduction and host-parasite interactions. Detailed phenotypic analysis has revealed that these kinases perform a role in adult worm movement, attachment, pairing and oviposition with pairing, worm tip velocity, ventral sucker attachment, and egg output reduced. Some of these outcomes (e.g., muscular movement and oviposition) might be interconnected. Intracellular signal transduction is complex and signalling pathways are also interconnected. Therefore, while it is possible that some of the observed phenotypic effects might result from indirect effects of kinase inhibition on associated pathways/mechanisms, the results of this paper indicate that PKCs and ERKs play an important role. Nevertheless, a goal of future research would be to define the importance of any indirect mechanisms on these phenotypes. Exploiting existing anti-cancer drugs that target protein kinases for the control of human parasites is an attractive approach, one that has been proposed previously for schistosomes [16]. Ras-Raf-MEK-ERK signalling represents a potential target in cancer therapy and several small molecule inhibitors of this pathway are being tested in clinical trials [81]. The PKC pathway also represents a potential target [82], [83]. Moreover, the insulin receptor, which signals through to PKC and ERK pathways has been proposed recently to be a viable vaccine target against schistosomes [84], [85]. Therefore, given the effects of global PKC and ERK inhibition on schistosome homeostasis and egg production, we propose that these pathways, including specific isotype analysis and isotype knockdown by RNAi, are investigated further as they represent potential anti-schistosome drug targets. Currently, we are studying PKC and ERK pathways in schistosomules, a stage that is PZQ-insensitive, to appreciate signalling by these kinases during host-parasite interactions and during early schistosome development in the human host.

## Supporting Information

Figure S1 **Negative control for confocal microscopy.** Representative confocal microscopy image of *S. mansoni* adult worm pair incubated with Alexa Fluor 488 secondary antibody (green) and rhodamine phalloidin (red) but not incubated in primary antibody. (A) red channel revealing actin staining and (B) green channel demonstrating lack of fluorescence in the absence of primary antibody. In each experiment a negative control was prepared to calibrate laser power and gain intensities, which were then kept constant for all observations. Images are of z-axis projections displayed in maximum pixel brightness mode.
(TIF)

Figure S2 **Long-term PMA exposure decreases PKC activation in adult** ***S. mansoni*** **.** Detection of phosphorylated (activated) *S. mansoni* PKCs after live adult worm pairs were exposed to 1 µM PMA or DMSO vehicle for 24 h. Protein homogenates (20 µg) were processed for Western blotting and blots probed with anti-phospho PKC (pan) (ζ Thr410) and anti-phospho PKC (pan) (βII Ser660) antibodies (Abs) in combination. Anti-actin antibodies were used to assess protein loading between samples. The blots shown are representative of those obtained from three experiments.
(TIF)

Figure S3 **Predicted PKCs in** ***S. mansoni*** **and tentative assignment of observed immunoreactive PKCs to Smp identifiers.** The relative gene expression data for cercariae (C), 3 h and 24 h schistosomules (3 h S and 24 h S), and adult worms (A) were extracted from GeneDB (www.genedb.org) [50].
(TIF)

Movie S1 **Control** ***S. mansoni*** **.** Adult worms were maintained in RPMI 1640 and were filmed after 1, 24, 48, 72, and 96 h.
(AVI)

Movie S2 **Inhibition of ERK induces separation of** ***S. mansoni*** **couples.** Adult worms were incubated in 50 µM U0126 and were filmed after 30 min.
(AVI)

Movie S3 **Inhibition of ERK induces separation of** ***S. mansoni*** **couples, reduces ventral sucker attachment, and affects worm motility.** Adult worms were incubated in 1, 5, 20 or 50 µM U0126 and were filmed after 1, 24, 48, 72 and 96 h.
(AVI)

Movie S4 **Inhibition of PKC induces separation of** ***S. mansoni*** **couples, reduces ventral sucker attachment, affects worm motility, and increases worm coiling.** Adult worms were incubated in 1, 5, 20 or 50 µM GF109203X and were filmed after 1, 24, 48, 72 and 96 h.
(AVI)

Movie S5 **Expulsion of blood from female** ***S. mansoni*** **in copula with a male.** Adult worms were treated with 20 µM GF109203X and were filmed at 1 h. Here the increased and sustained worm coiling likely caused blood to be expelled from the female worm.
(AVI)

Movie S6 **PMA induces separation of** ***S. mansoni*** **couples, reduces ventral sucker attachment, and affects worm motility.** Adult worms were incubated in 1 µM PMA and were filmed after 1, 24, 48, 72 and 96 h.
(AVI)
