# Supplementary figures and images for: Protein Kinase C and Extracellular Signal-Regulated Kinase Regulate Movement, Attachment, Pairing and Egg Release in Schistosoma mansoni
Source: PLoS Negl Trop Dis. 2014 Jun 12;8(6):e2924. doi: 10.1371/journal.pntd.0002924 (PMC4055629; doi:10.1371/journal.pntd.0002924)

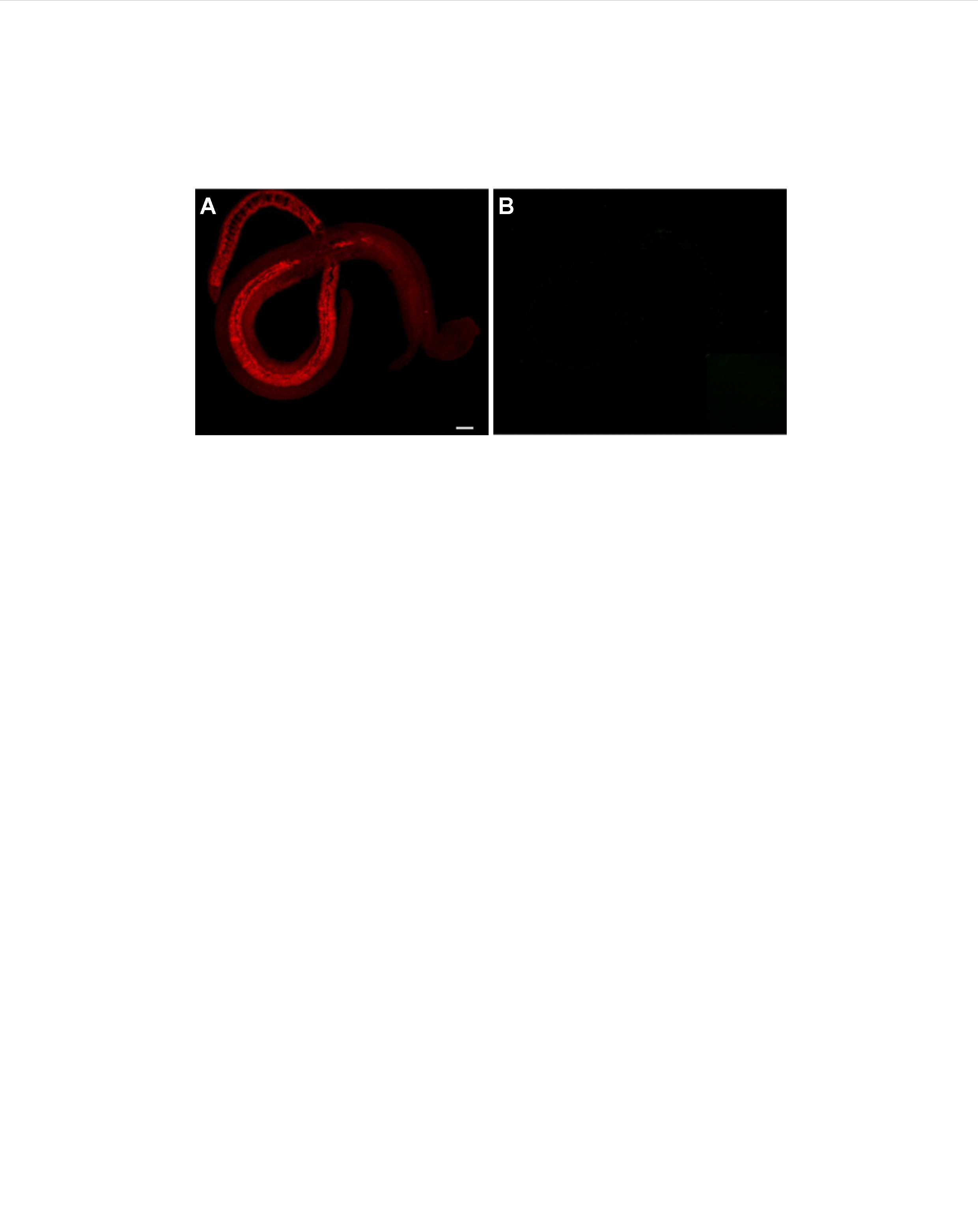

Supplement: Figure S1 — Negative control for confocal microscopy. Representative confocal microscopy image of S. mansoni adult worm pair incubated with Alexa Fluor 488 secondary antibody (green) and rhodamine phalloidin (red) but not incubated in primary antibody. (A) red channel revealing actin staining and (B) green channel demonstrating lack of fluorescence in the absence of primary antibody. In each experiment a negative control was prepared to calibrate laser power and gain intensities, which were then kept constant for all observations. Images are of z-axis projections displayed in maximum pixel brightness mode. (TIF) [file pntd.0002924.s001.tif]

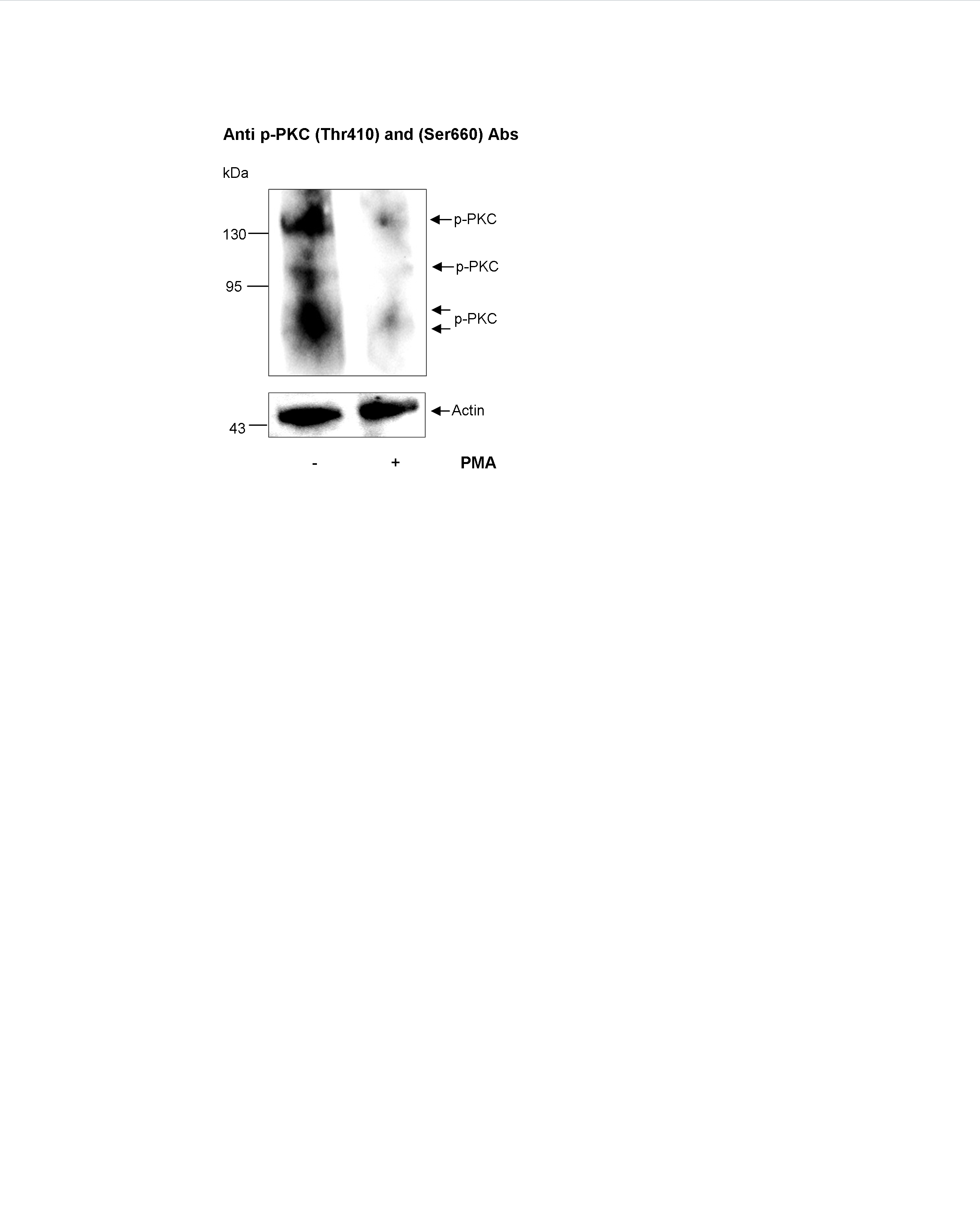

Supplement: Figure S2 — Long-term PMA exposure decreases PKC activation in adult S. mansoni . Detection of phosphorylated (activated) S. mansoni PKCs after live adult worm pairs were exposed to 1 µM PMA or DMSO vehicle for 24 h. Protein homogenates (20 µg) were processed for Western blotting and blots probed with anti-phospho PKC (pan) (ζ Thr410) and anti-phospho PKC (pan) (βII Ser660) antibodies (Abs) in combination. Anti-actin antibodies were used to assess protein loading between samples. The blots shown are representative of those obtained from three experiments. (TIF) [file pntd.0002924.s002.tif]

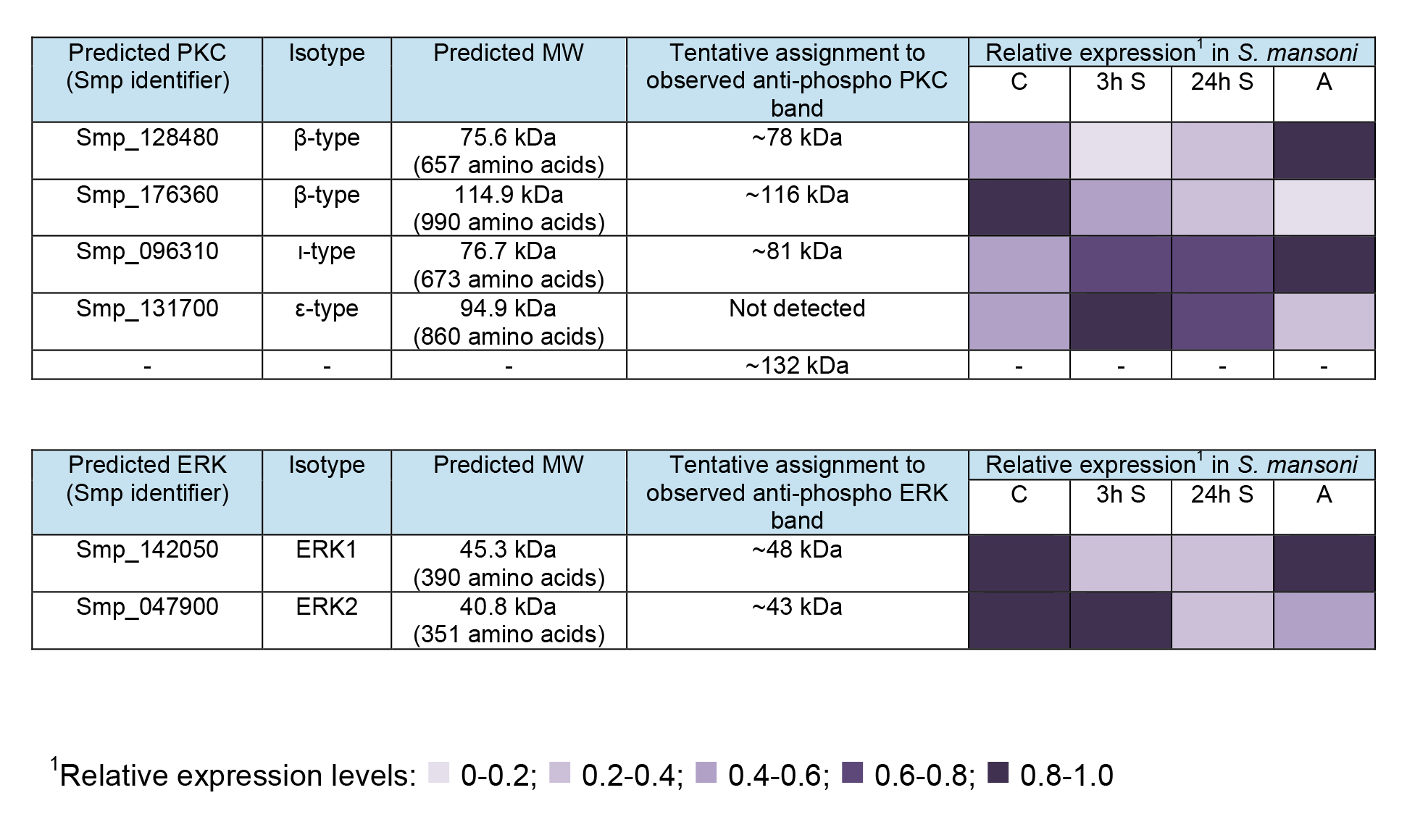

Supplement: Figure S3 — Predicted PKCs in S. mansoni and tentative assignment of observed immunoreactive PKCs to Smp identifiers. The relative gene expression data for cercariae (C), 3 h and 24 h schistosomules (3 h S and 24 h S), and adult worms (A) were extracted from GeneDB (www.genedb.org) [50]. (TIF) [file pntd.0002924.s003.tif]
